# Homolytic X‐H Bond Cleavage at a Gold(III) Hydroxide: Insights into One‐Electron Events at Gold

**DOI:** 10.1002/chem.202200599

**Published:** 2022-05-31

**Authors:** Silène Engbers, Isaac F. Leach, Remco W. A. Havenith, Johannes E. M. N. Klein

**Affiliations:** ^1^ Molecular Inorganic Chemistry Stratingh Institute for Chemistry Faculty of Science and Engineering University of Groningen Nijenborgh 4 9747 AG Groningen (The Netherlands; ^2^ Zernike Institute for Advanced Materials Faculty of Science and Engineering University of Groningen Nijenborgh 4 9747 AG Groningen (The Netherlands; ^3^ Ghent Quantum Chemistry Group Department of Chemistry Ghent University Ghent 9000 Gent Belgium

**Keywords:** computational chemistry, gold, oxidations, proton coupled electron transfer, reaction mechanisms

## Abstract

C(sp^3^)‐H and O−H bond breaking steps in the oxidation of 1,4‐cyclohexadiene and phenol by a Au(III)‐OH complex were studied computationally. The analysis reveals that for both types of bonds the initial X−H cleavage step proceeds via concerted proton coupled electron transfer (cPCET), reflecting electron transfer from the substrate directly to the Au(III) centre and proton transfer to the Au‐bound oxygen. This mechanistic picture is distinct from the analogous formal Cu(III)‐OH complexes studied by the Tolman group (*J. Am. Chem. Soc*. **2019**, 141, 17236–17244), which proceed via hydrogen atom transfer (HAT) for C−H bonds and cPCET for O−H bonds. Hence, care should be taken when transferring concepts between Cu−OH and Au−OH species. Furthermore, the ability of Au−OH complexes to perform cPCET suggests further possibilities for one‐electron chemistry at the Au centre, for which only limited examples exist.

## Graphical abstract

**The mechanism of homolytic X‐H bond cleavage** by a Au(III)‐OH complex was investigated computationally. Both C−H and O−H bonds are cleaved via a concerted proton coupled electron transfer mechanism (cPCET) rather than a hydrogen atom transfer mechanism (HAT). The results suggest that one‐electron events at gold may be more accessible than anticipated.

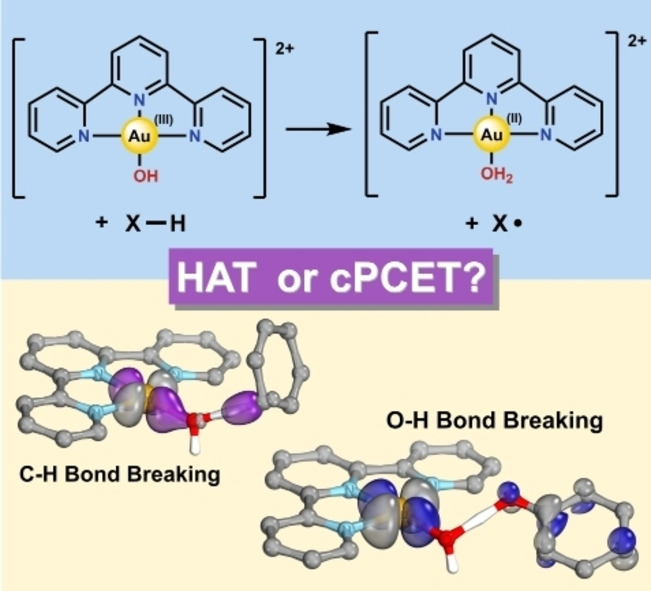

Until recently, the known reactivity of Au(III) complexes featuring ligands bound through an oxygen atom (Au(III)‐O complexes), including Au(III)‐hydroxide, Au(III)‐alkoxides, Au(III)‐peroxide and Au(III)‐alkyl peroxides, was mostly limited to ligand exchange and oxygen atom transfer (OAT) reactions.^[1]^ Although Au(III)‐O complexes have been previously suggested to oxidize C−H bonds in catalysis,^[2]^ the McDonald group recently reported the ability of a structurally well‐defined Au(III)‐hydroxide complex to cleave weak C−H and O−H bonds stoichiometrically, through a proton coupled electron transfer (PCET) process.^[3]^ Mechanistically, OAT entails the transfer of the gold‐bound oxygen atom to a phosphine as a two electron oxidation, thereby forming a Au‐hydride without any change in the formal oxidation state of Au (Scheme 1a). In contrast to this, PCET describes the abstraction of a proton and single electron from a substrate, thereby forming the one‐electron reduced Au(II)‐OH_2_ species (Scheme 1b). This one‐electron reduction is remarkable considering that mononuclear Au(II) species are known to be highly unstable.^[4]^

Several distinct mechanistic scenarios exist within the PCET umbrella term. These have, however, been at times ill‐defined in the literature.^[5]^ We will use the following definitions: the term PCET includes both step‐wise and concerted mechanisms. Within the concerted regime, two variants are possible: hydrogen atom transfer (HAT) and concerted proton coupled electron transfer (cPCET). Whereas HAT requires that the proton and electron move as a true hydrogen atom (together), cPCET describes a process in which the proton and electron move separately but simultaneously (Scheme 2).

**Scheme 1:**
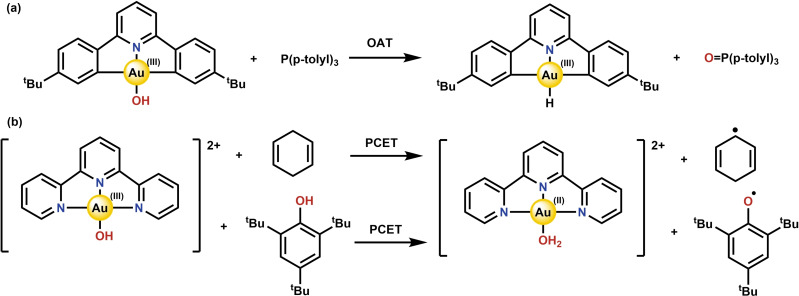
(a) OAT of a Au‐hydroxide complex as reported by the Bochmann group (ref. [1c]), (b) PCET of a Au‐hydroxide as reported by the McDonald group (ref. [3]).

**Scheme 2:**
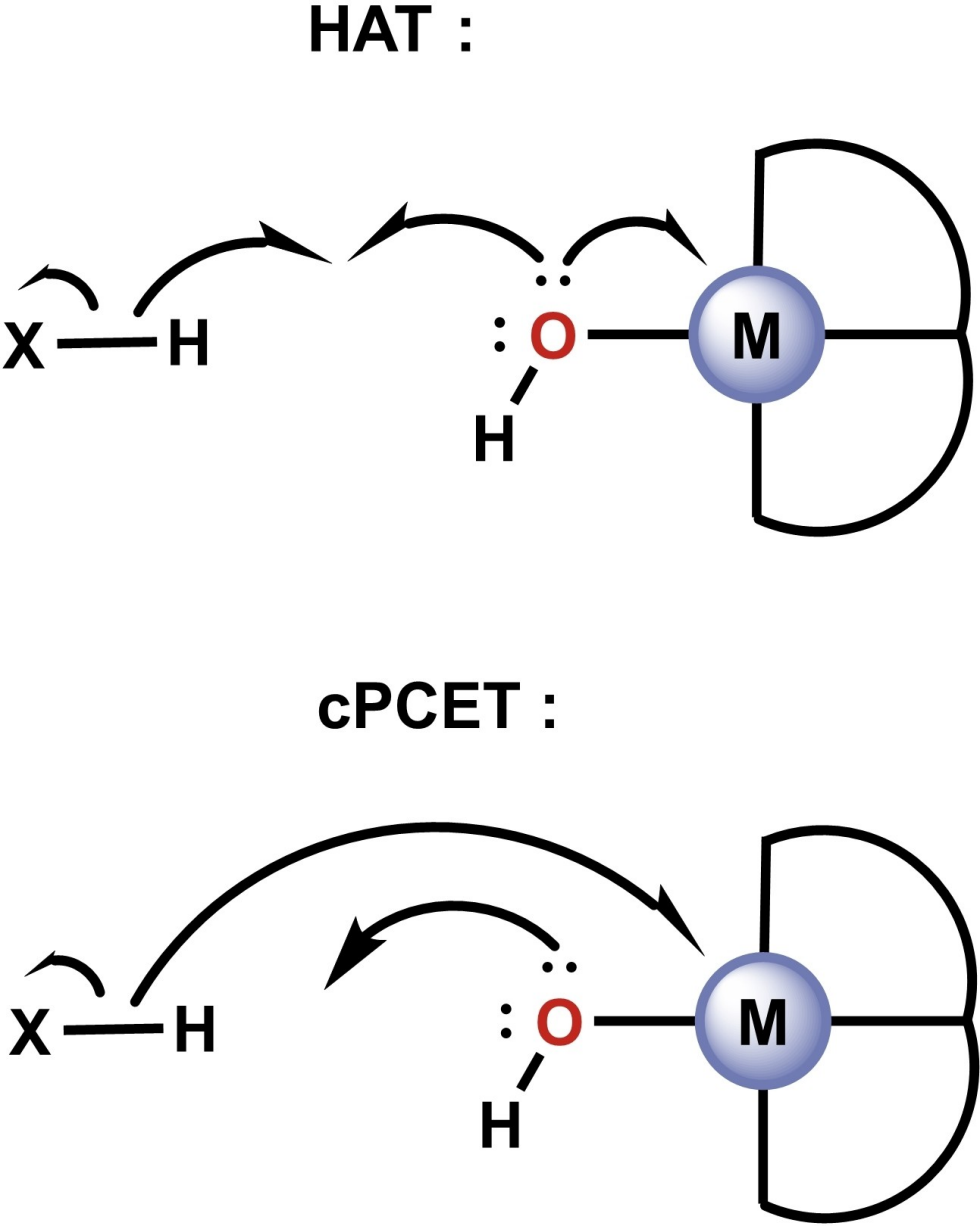
Representations of the electron flow in HAT and cPCET mechanisms.

The complex [Au(OH)(terpy)](ClO_4_)_2_, where terpy is 2,2’ : 6’,2‐terpyridine, is capable of performing PCET reactions with 1,4‐cyclohexadiene (CHD), 9,10‐dihydroanthracene (DHA), and a variety of electron rich phenols.[^3^, ^6^] These reactions were proposed to proceed via HAT based on kinetic data.^[3]^ We note that the isoelectronic Cu(III)‐hydroxide complex, (N^N^N)Cu(OH) where (N^N^N) is *N*,*N*’‐bis(2,6‐diisopropylphenyl)‐2,6‐pyridinedicarboxamide, has similarly been shown to perform PCET with DHA and phenols.[^5h^, ^7^] A computational study indicated that, in the case of this Cu‐hydroxide, C−H and O−H bond cleavage occur through different mechanisms. Whereas HAT is operative for C−H bond cleavages, O−H bond breaking occurs through a cPCET mechanism (Scheme 3).^[5h]^

**Scheme 3:**
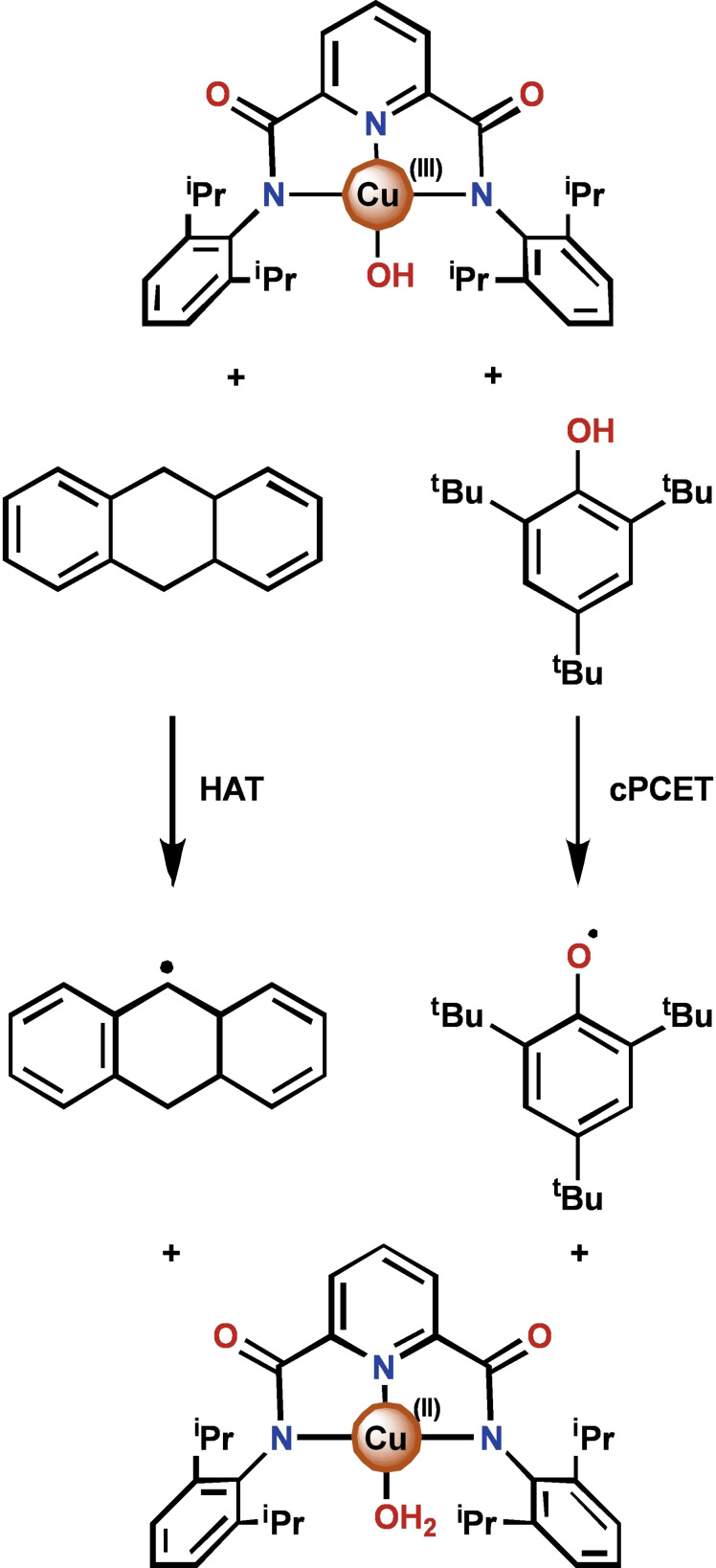
PCET reactivity of (N^N^N)Cu(OH) with DHA and 2,6‐ditertbutylphenol as reported by the Tolman group (ref. [5h]).

Considering the subtle variation in mechanisms for the Cu‐hydroxide with C−H and O−H bonds, we opted to further investigate the X−H bond cleavage mechanism(s) of [Au(OH)(terpy)]^2+^ with CHD and phenol using Density Functional Theory and compare it to the (N^N^N)Cu(OH) case. For a justification of the substrate choice see Supporting Information section 3. Using the intrinsic bond orbital (IBO) formalism,[^5a^, ^5g^, ^5h^, ^8^] and analysing the change in projected dipole moment along the reaction coordinate,[^5h^, ^9^] we were able to differentiate between HAT and cPCET mechanisms.

We began our investigation by optimizing transition states for both reactions using PBE0‐D3(BJ)/def2‐SVP/(c)PCM(DMF)^[10]^ (for full computational details see Supporting Information section 1). Although [Au(OH)(terpy)]^2+^ and its substrates, CHD and phenol, are all closed‐shell species, the products of the PCET reactions of interest, [Au(OH_2_)(terpy)]^2+^ and the cyclohexadienyl or phenoxyl radical, are all open‐shell species. Hence, the reaction must proceed through a broken symmetry transition state, and the resulting spin contamination can be rationalized by the appearance of spin density (Figure 1). The formation of spin density on the Au centre and substrates at the obtained transition states confirms that, for both reactions, PCET is indeed operative rather than proton or hydride transfer. After spin purification by the Yamaguchi spin purification scheme,[^7a^, ^11^] the bond breaking free energies of activation were found to be 11.1 and 9.3 kcal mol^−1^ for the reactions with CHD and phenol, respectively. Furthermore, the change in free energy of the reactions were found to be −8.2 and 7.3 kcal mol^−1^, respectively. It should be noted that although the reaction with phenol is endothermic, the Au(II) intermediate is transient and full decomposition is observed, thereby providing the thermodynamic driving force for the reaction.

**Figure 1:**
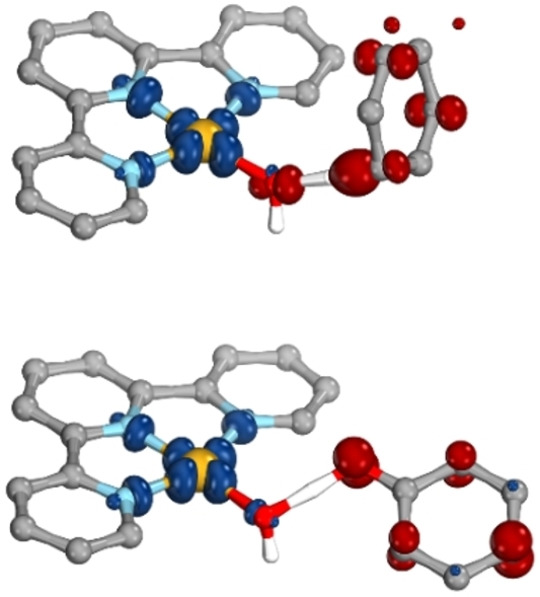
Spin density isosurface of the transition state of the reaction with CHD (top), and the reaction with phenol (bottom). Calculated with PBE0‐D3(BJ)/def2‐SVP/(c)PCM(DMF). All hydrogen atoms, apart from H_OAu_, have been omitted for clarity. Blue and red indicate +/− 0.009e, respectively.

We recognize that the choice of functional can have an impact on the nature of the transformation. PBE0, featuring 25 % exact exchange, was selected for this study. However, increasing or decreasing the amount of exact exchange of this functional was found to influence the extent of electron transfer. Namely, when decreasing the amount of exact exchange to 0 % the reaction resembled a closed shell hydride transfer and increasing the exact exchange to 100 % provided a simple electron transfer reaction (see Supporting Information section 2.1 for more details).

To validate our method, CASSCF calculations were performed at the transition state structures and the unpaired electron density was analysed. Results are consistent with homolytic bond cleavage (see Supporting Information section 2.2), thus suggesting an open shell reaction and deeming the use of PBE0 to provide an appropriate representation of the nature of the reaction.

To distinguish which subcategory of PCET is operative for the reaction with CHD, the transformations of the intrinsic α‐ and β‐spin orbitals of the C−H σ‐bond were followed along the reaction coordinate (Figure 2). The α‐spin orbital of the C−H bond (A, purple) clearly transforms into the Au 5d_x_^2^_‐y_^2^ orbital, whereas the β‐spin orbital (B, green) transforms into a π‐orbital of the cyclohexadienyl radical. Thus, a cPCET mechanism as described in Scheme 2 is operative. Further small electronic structure changes of the Au−O bond and oxygen lone pair can be found in Figure S5. Of particular interest is the α‐spin orbital of the Au−OH bond (B, red), which has almost entirely transformed before the OH lone pair (A, blue) and Au−O (C, green) β‐spin orbitals begin to change. Orbital changes along the reaction coordinate can thus be considered to be rather gradual.

**Figure 2:**
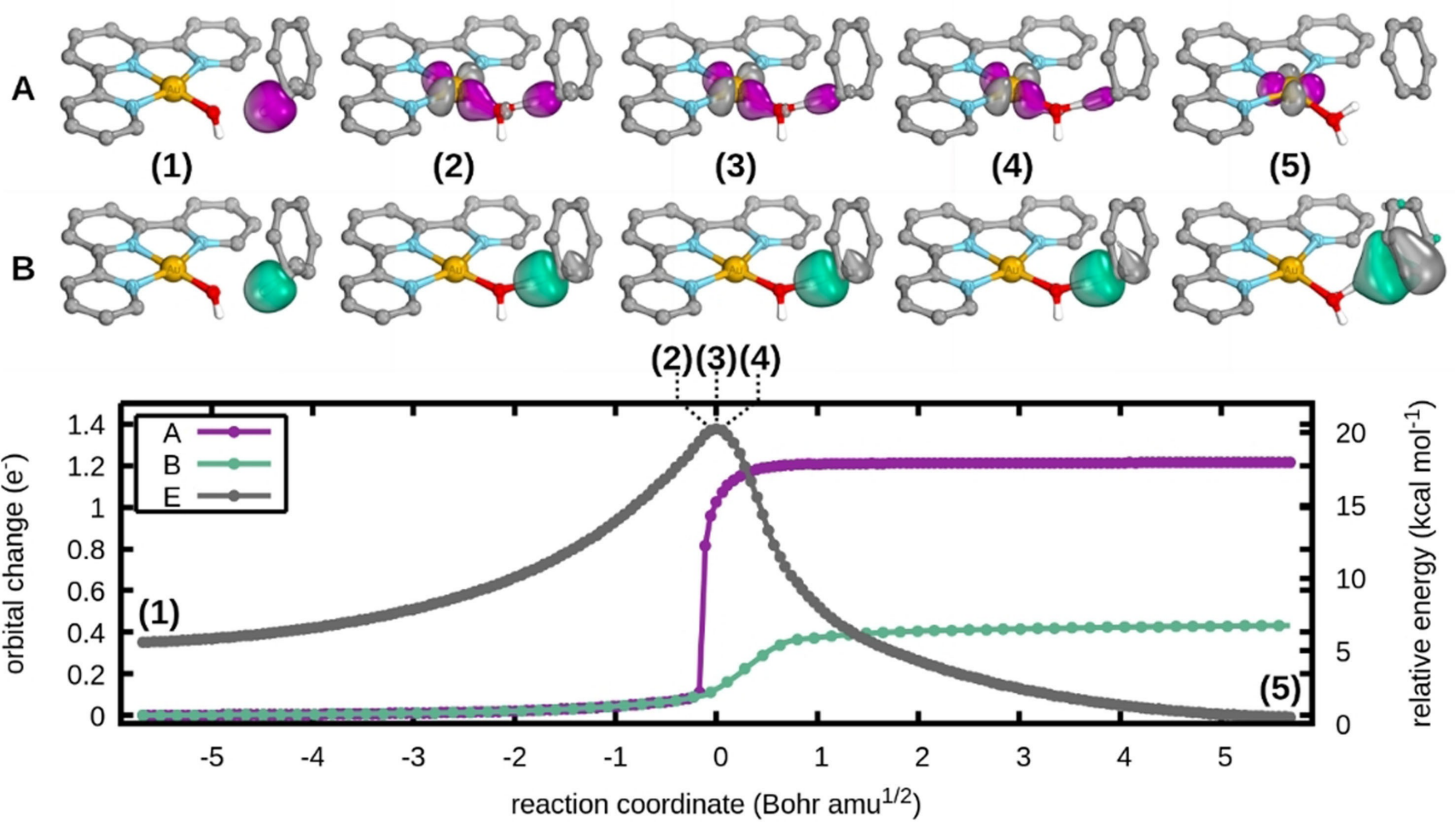
Electron flow analysis (A and B) and relative energies reference to the fully relaxed separated reactants (E) of the reaction with CHD showing the orbital transformations of the C−H bond. All hydrogens apart from H_CHD_ and H_OAu_ of the reactant have been omitted for clarity. Calculated with PBE0‐D3(BJ)/def2‐SVP/(c)PCM(DMF).

Similarly, the relevant IBO transformations were followed for the O−H bond breaking reaction with phenol (Figure 3). In this case, both the α‐ and β‐spin orbitals of the O−H σ‐bond transform into the lone pair on the phenoxy radical (B, green and C, red), and it is actually an α‐spin orbital from a π‐bond of the aromatic system that transforms to the Au 5d_x_^2^_‐y_^2^ spin orbital (A, blue). This still indicates that cPCET is operative, as an electron is transferred directly from the substrate to the Au centre, rather than travelling with the proton to the hydroxide moiety. However, the orbital transformations deviate slightly from the classical description outlined in Scheme 2 as the electron moving to the Au centre originates from the π‐system of the substrate rather than the O−H σ‐bond, which is well in line with the expected reactivity for phenol substrates.^[12]^

**Figure 3:**
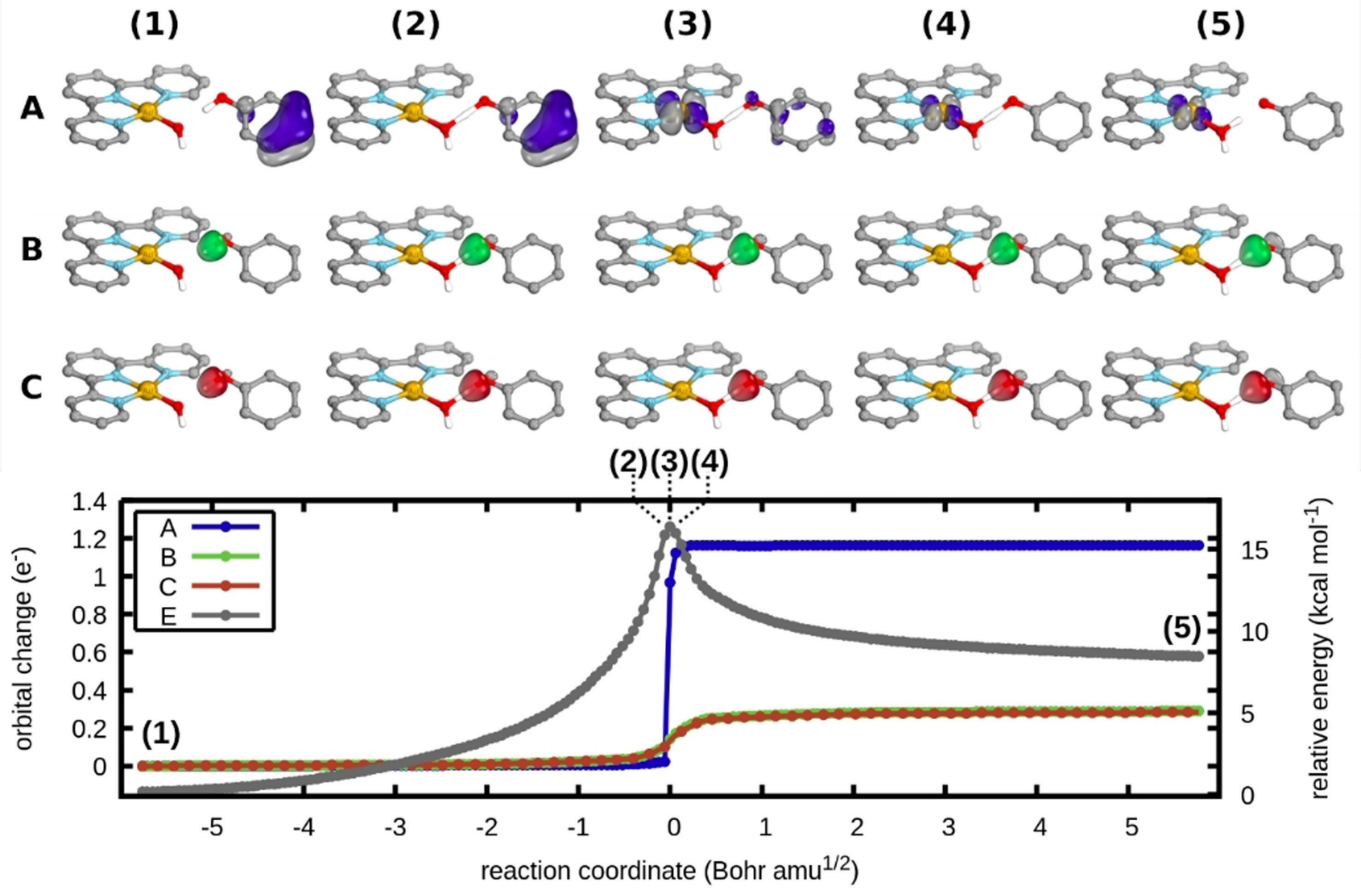
Electron flow analysis (A, B and C) and relative energies reference to the fully relaxed separated reactants (E) of the reaction with phenol showing the orbital transformations of the O−H bond (B, C) and the oxidation of the metal centre by the aromatic system (A). All hydrogens apart from H_OPh_ and H_OAu_ of the reactant have been omitted for clarity. Calculated with PBE0‐D3(BJ)/def2‐SVP/(c)PCM(DMF).

It should also be noted that both the α‐ and β‐spin orbitals of the Au−OH bond transform into the Au−OH_2_ bond of the product (Figure S6), reflecting simple proton transfer between the two sites. Orbital changes associated with electron transfer are also abrupter for phenol than CHD. This can be seen by the manner in which IBOs for phenol transform more simultaneously along the reaction coordinate. The derived curly arrow mechanisms of both reactions can be found in section 5 of the Supporting Information.

To further verify that both reactions proceed via a cPCET mechanism, the change in dipole moment projected onto the AuO−H‐X axis was followed along the reaction coordinate (Figure 4).^[5h]^ In the case of a HAT reaction, minimal change in the projected dipole moment should be observed as the proton and electron move as one neutral moiety towards the Au−O oxygen atom. However, for cPCET, the electron and proton move simultaneously but separately, one travelling toward the oxygen atom and the other towards the Au centre. This charge separation has an increased influence on the total dipole moment along the AuO−H‐X axis. As a general guideline, changes below 5 Debye are considered to fall within the HAT category whereas changes between 6 and 25 Debye are considered cPCET.^[5h]^ In our case, the changes in projected dipole moment were found to be 12 and 22 Debye for CHD and phenol, respectively, further supporting their classification as cPCET reactions.

**Figure 4:**
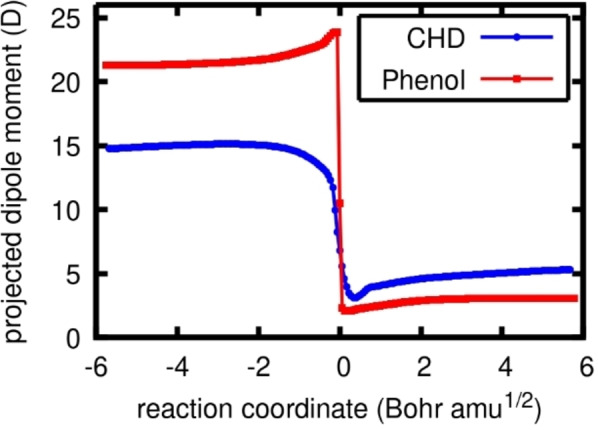
Projected dipole moments for the reactions with CHD (blue) and phenol (red), along the IRC, calculated with PBE0‐D3(BJ)/def2‐SVP/(c)PCM(DMF).

The significant difference in projected dipole moment change can be attributed to the larger distance travelled by the electron in the case of phenol, as it originates from the π‐system rather than the C−H σ‐bond. Furthermore, we notice that the plots in Figure 4 differ in shape as that of CHD (blue) is smoother and that of phenol (red) more abrupt at the transition state. This can be related to the more abrupt electron transfer of phenol compared to CHD, as observed in the orbital diagrams.

We thus conclude that, in contrast to the analogous (N^N^N)Cu(OH) complex reported by the Tolman group, which cleaves C−H bonds via HAT and O−H bonds via cPCET,^[5h]^ [Au(OH)(terpy)]^2+^ cleaves both via cPCET mechanisms. Although this is a striking difference, we are cautious of drawing generalizations regarding the two transitions metals due to the overall charge present on the Au complex which is not present in the Cu case. As these are isolated examples, we encourage new PCET reactions to be studied individually, on a case‐by‐case basis. However, we are hopeful that, when more data becomes available, an interesting trend will arise for the coinage metals.

More generally, the observation that Au−OH complexes are capable of cPCET reactions suggests that one‐electron chemistry at Au may be more accessible than anticipated. Although mononuclear Au(II) complexes are known to be rather unstable and Au is thought to be unwilling to perform one‐electron chemistry, recent examples of photo redox catalysis suggest the involvement of Au(II) intermediates.^[13]^ Hence, we can anticipate new directions for Au(II) chemistry in the future.

## Conflict of interest

The authors declare no conflict of interest.

## Supporting information

As a service to our authors and readers, this journal provides supporting information supplied by the authors. Such materials are peer reviewed and may be re‐organized for online delivery, but are not copy‐edited or typeset. Technical support issues arising from supporting information (other than missing files) should be addressed to the authors.

Supporting Information

## Data Availability

The data that support the findings of this study are available in the supplementary material of this article.
