# Supplementary material for: Homolytic X‐H Bond Cleavage at a Gold(III) Hydroxide: Insights into One‐Electron Events at Gold
Source: Chemistry. 2022 May 31;28(40):e202200599. doi: 10.1002/chem.202200599 (PMC9401072; doi:10.1002/chem.202200599)
Supplement: Supplementary file 1 — Supporting Information [file CHEM-28-0-s001.pdf]

Chemistry–A European Journal

Supporting Information

Homolytic X-H Bond Cleavage at a Gold(III) Hydroxide: Insights into One-Electron Events at Gold

Silène Engbers, Isaac F. Leach, Remco W. A. Havenith, and Johannes E. M. N. Klein*

Table of Contents

1. Computational Details	S2
2. Method Justification	S3
3. Substrate Justification	S5
4. Additional Figures	S6
5. Derived Curly Arrow Mechanisms	S7
6. Cartesian Coordinates	S8
7. Intrinsic Bond Orbital Changes	S14
8. Dipole Projection	S22
9. References	S30

1. Computational Details

1.1 Methods

All geometry optimizations were performed in Gaussian 16 rev C.01[1] using Density Functional Theory. These calculations used the PBE0[2] functional and the def2-SVP[3] basis set (specified via the Basis Set Exchange[4]). A pseudo potential was used for the Au atom.[5] Dispersion effects were accounted for by Grimme's D3 model,[6] with Becke Johnson damping[7]. Solvent effects were modelled implicitly with the Polarization Continuum Model (PCM),[8] with *N*,*N*-dimethylformamide (DMF) specified as the solvent. Increased accuracy of the two-electron integrals and the integration grid were requested via the *acc2e=16* and *Grid=UltraFine* keywords, respectively. Default convergence criteria were used. Where appropriate, Kohn-Sham wavefunctions were checked for internal stability with the Stable=Opt keyword, and in the case that instabilities (i.e., broken symmetry solutions) were found, the structures were reoptimized.

<u>Example input line</u>:
#p Integral(acc2e=16,Grid=UltraFine) SCRF(PCM,Solvent=n,n-DiMethylFormamide) pop=always UPBE1PBE/GenECP EmpiricalDispersion=GD3BJ SCF=XQC OPT(MaxStep=5) Freq=NoRaman

Frequency analyses confirmed the nature of all stationary points and intrinsic reaction coordinate calculations ensured all reactants were connected to product structures via single transition states on the potential energy surface. Free energies include the concentration change term RTln(24.5) = 1.89 kcal mol^{-1}.[9]

1.2 Spin Purification

Spin purified energies of broken symmetry structures can be found in Table S1. Spin purification was performed in order to remove triplet-spin contamination according to the Yamaguchi spin purification scheme,[10] where BS = broken symmetry:

$$E^{S_0} = \frac{2E^{BS} - < S^2 >^{BS} E^{T_1}}{2 - < S^2 >^{BS}}$$

Table S1. Energies of the broken symmetry species after spin purification

**Species**	**Spin Purified SCF Energy**
TS_{CHD}	-1185.20577129448
IC_{CHD}	-1185.23816851957
TS_{PhOH}	-1259.13921953256
IC_{PhOH}	-1259.15302588799

1.3 Intrinsic Bond Orbitals

To understand the nature of the electronic transformation during the reaction, electron flow analyses[11] were performed on each of the set of geometries from the IRC calculations (*vide supra*). These calculations were performed in ORCA 4.2.1,[12] using the PBE0[2] functional and the def2-SVP[3] basis set. Dispersion effects were accounted for by Grimme's D3 model,[6] with Becke Johnson damping.[7]. The conductor-like Polarization Continuum Model (cPCM)[13] was used to implicitly model solvent effects, with *N*,*N*-dimethylformamide (DMF) specified as the solvent. A modified (van der Waals Gaussian) cavity was used for cPCM. The *RIJCOSX* approximation was used to accelerate the evaluation of the two-electron integrals, with Weigend's universal fitting basis set (*def2/J*).[14] Increased accuracy of the integration grid was requested via the *Grid6 NoFinalGrid* keywords. An SCF energetic convergence criterion of 10^{-8} Hartree was specified (*TightSCF*).

Example input line:
! UKS PBE0 D3BJ def2-SVP def2/J RIJCOSX Grid6 GridX6 NoFinalGrid CPCM(DMF) SlowConv SOSCF TightSCF
%cpcm
SurfaceType vdw_gaussian
end

Localization of the Kohn-Sham orbitals was performed in IboView[15] (freely available at www.iboview.org) using *exp2*. The resulting IBOs were visualized in IboView to enclose 80% of their electron density.

1.4 Projected Dipole Moments

Following the method of Mandal *et al.*,[16] the dipole moment was projected onto the substrate-H-Au coordinate and plotted against the total reaction coordinate (as computed in the Gaussian IRC calculation). The dipole moment was taken from a series of single point calculations on the IRC trajectory – see section 1.3 for details. The following formula was used to project the total dipole moment (**a**) onto the substrate-H-O_{Au} coordinate (**b**), thereby yielding the projected dipole moment (**a'**):

$$\boldsymbol{a}' = \frac{\boldsymbol{a} \cdot \boldsymbol{b}}{\boldsymbol{b} \cdot \boldsymbol{b}} \boldsymbol{b}$$

The substrate-H-O_{Au} coordinate was defined as the vector connecting oxygen atom #33 to oxygen atom #32 in the case of phenol (PhOH) and from carbon atom #34 to oxygen atom #2 for cyclohexadiene (CHD).

See section 8 for the dipole values before and after projection.

2. Method Justification

2.1 Sensitivity to the Amount of Exact Exchange

As noted, we do observe some dependency of the electronic structure on the chosen DFT functional and its % of HF exchange. The standard hybrid PBE0 functional uses 25% HF exchange. We modified this to 0% (PBE) and 100% (PBE100). The calculated Kohn-Sham wavefunctions show some qualitative differences, which can be understood via inspection of their spin density plots (Figures S1 and S2).

With PBE, the spin polarization is greatly reduced in the reaction with PhOH. With CHD, no spin polarization is observed in the TS (Figure S1b); $\rho_\alpha = \rho_\beta$ i.e., the solution is closed shell. Examination of the IBOs reveals that the proton is being transferred along with an α-electron (Figure S2a) and a β-electron (Figure S2b), consistent with an overall hydride transfer mechanism.

With PBE100, increased spin density is observed in the reactions with both CHD and PhOH. Even the reactant complexes (RC, Figure S3a and S3d) show a large accumulation of spin density, indicating electron transfer has already occurred. This points towards a stepwise mechanism, with electron transfer followed by proton transfer.

The PBE0 functional we chose is of course a balance between these two scenarios. We were nonetheless motivated to further validate our conclusions (of a PCET mechanism) with a wavefunction-based method, namely CASSCF (see section 2.2).

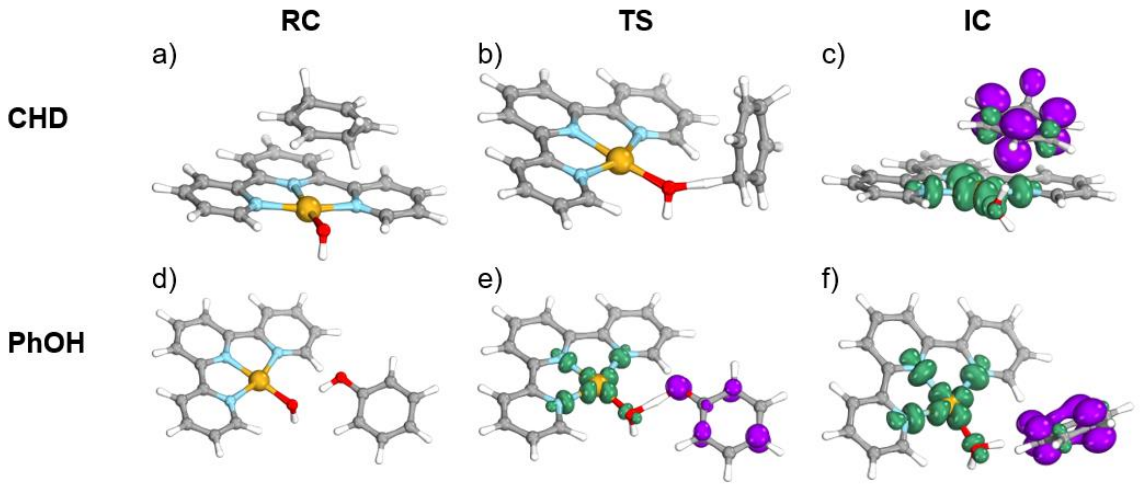

Figure S1: Spin density plots of the reactant complex (RC), transition state (TS) and intermediate complex (IC) of the reactions with CHD (a-c) and PhOH (d-f), calculated with PBE-D3(BJ)/def2-SVP/cPCM(DMF) in ORCA 4.2.1, positive spin density is depicted in green and negative spin density in purple (isosurface 0.004). Rendered with IboView.

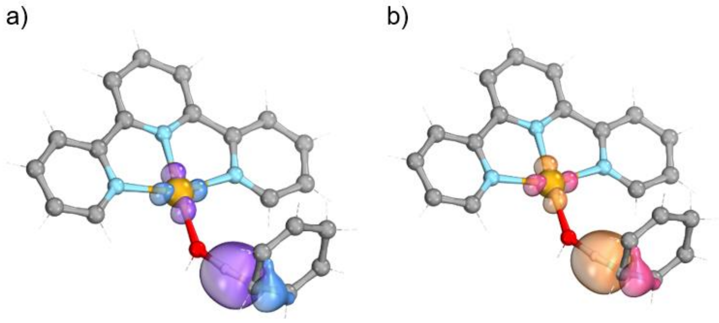

Figure S2: Alpha (a) and beta (b) IBOs of the transition state of the reaction with CHD calculated with PBE-D3(BJ)/def2-SVP/cPCM(DMF) in ORCA 4.2.1. Rendered in IboView with the isosurfaces drawn to enclose 80% of each orbital’s electrons density.

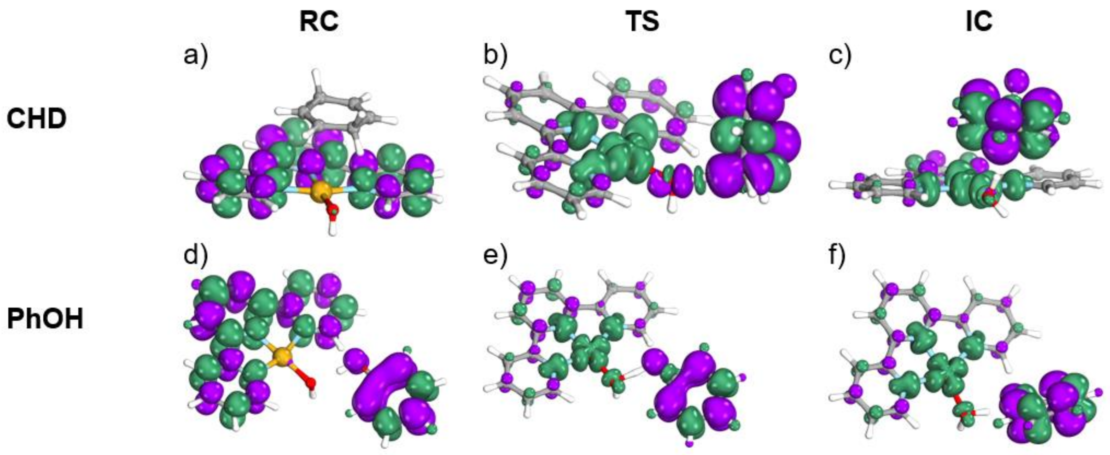

Figure S3: Spin density plots of the reactant complex (RC), transition state (TS) and intermediate complex (IC) of the reactions with CHD (a-c) and PhOH (d-f), calculated with PBE100-D3(BJ)/def2-SVP/cPCM(DMF) in ORCA 4.2.1, positive spin density is depicted in green and negative spin density in purple (isosurface 0.004). Rendered with IboView.

2.2 CASSCF Calculations

Given the observed sensitivity of the results to the portion of HF exchange in the DFT functional (see section 2.1), we performed CASSCF calculations with GAMESS-UK,[17] at the optimized PBE0-D3(BJ)/def2-SVP/PCM(DMF) transition state geometries. These calculations started from Unrestricted Hartree-Fock (UHF) natural orbitals using the def2-SVP basis set, following Pulay's UNO-CAS procedure.[18] Natural orbitals with occupation number (η) between $0.1 < \eta < 1.9$ were selected for the active space. We analyzed the unpaired electron density.[19] The results are summarized in Table S2 and are consistent with homolytic C-H bond cleavage.

Table S2: Effective number of unpaired electrons on the complex (Au(ligand)-OH) and substrate (CHD or PhOH) fragments.

**Fragment**	**CHD**<br>CASSCF(6,6)	**PhOH**<br>CASSCF(12,12)
Complex	0.9201	1.9498
Substrate	1.3098	1.3144

3. Substrate Choice

3.1 DHA vs. CHD

We note that, in the present study we investigate the C-H bond breaking of CHD, one of the substrates investigated experimentally by the McDonald group for $[(terpy)Au(OH)]^{2+}$.[20] However, in the case of (N^N^N)Cu(OH) reported by the Tolman group the reaction with dihydroanthracene (DHA) was investigated computationally.[16] These two substrates are commonly used in C-H bond breaking reactions and are also often used interchangeably. Moreover, a previous study by Klein et al. demonstrates that (N^N^N)Cu(OH) reacts with both CHD and DHA similarly.[10h] For additional validation, single point calculations were performed on these previously reported transition state geometries[10h] using the method outlined in section 1.3 and their IBOs were then analyzed (Figure S4). In both cases, the α-spin orbital of the C-H σ-bond transforms onto the Cu-bound oxygen atom to form an O-H σ-bond, indicating that a HAT mechanism is operative for both substrates. Hence, we can conclude that our mechanistic comparison between the Cu-OH and Au-OH complexes remains valid regardless of the difference in substrates.

a) b)

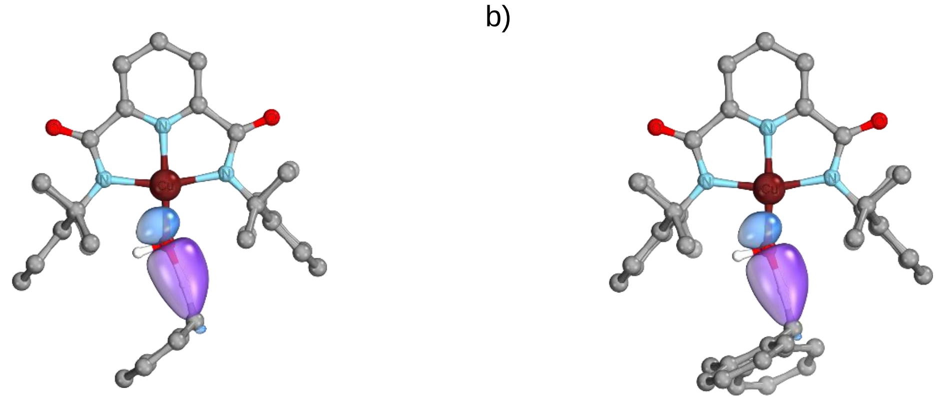

Figure S4: IBOs of the transition states of the reactions of (N^N^N)Cu(OH) with CHD (a) and DHA (b). Geometries were obtained from ref. [10h] and single point calculations were performed with PBE0-D3(BJ)/def2-SVP/cPCM(DMF) in ORCA 4.2.1. Rendered in IboView with the isosurfaces drawn to enclose 80% of each orbital's electrons density. All hydrogens apart from $H_{CHD/DHA}$ and H_{OCu} of the reactant have been omitted for clarity.

3.2 2,6-di*tert*butyl substituted phenols vs. PhOH

The O-H bond breaking reactions by both $[(terpy)Au(OH)]^{2+}$[20] and (N^N^N)Cu(OH)[16] were experimentally investigated for the reaction with 2,6-di*tert*buyl substituted phenols. In contrast, in our present computational study we employ PhOH. In another study by the Tolman group, a range of phenols, including phenol itself, have been shown to be consistently oxidized by (N^N^N)Cu(OH) via a cPCET mechanism.[21] Taking this into consideration, we judge it unlikely that the change in substrate will lead to a different mechanism for the $[(terpy)Au(OH)]^{2+}$ complex studied here. Hence, the simplest substrate (phenol) was chosen for convenience and our comparison between the two metal complexes should remain valid regardless of this choice.

4. Additional Figures

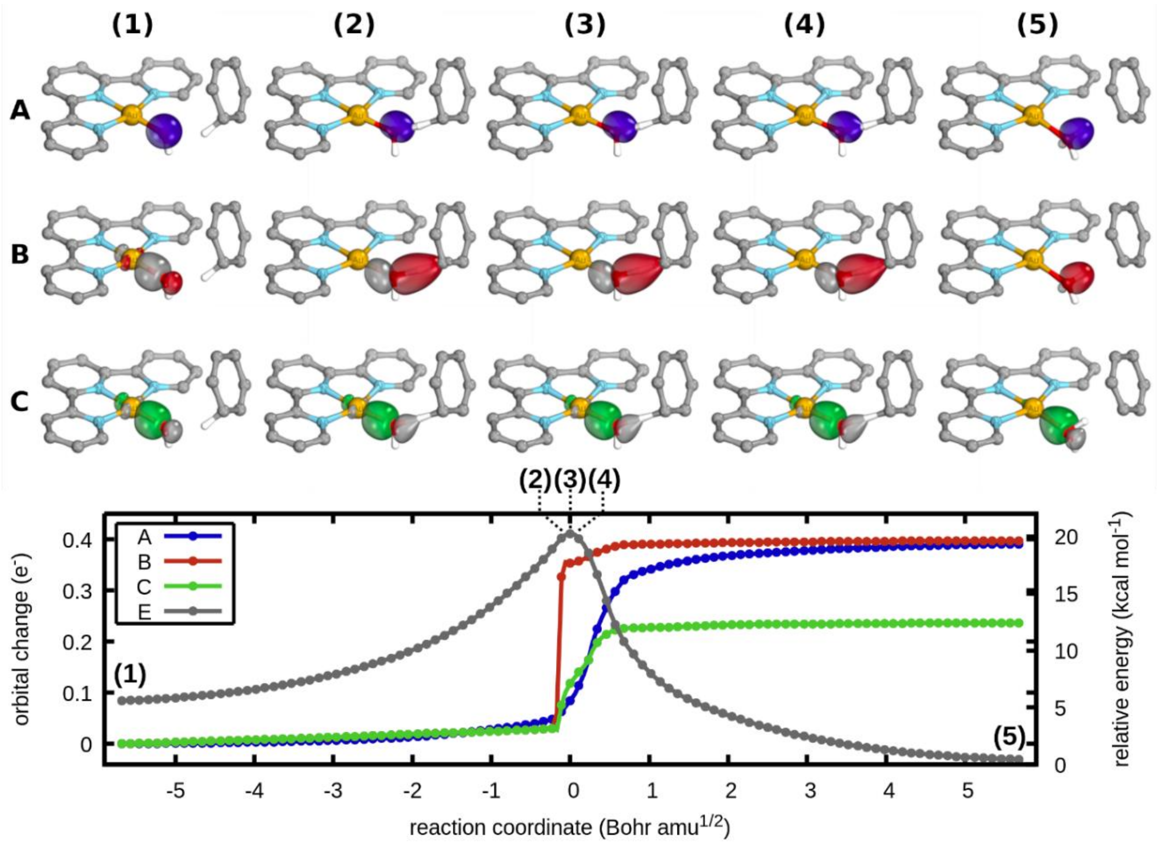

Figure S5: Electron flow analysis (A, B, and C) and relative energies reference to the fully relaxed separated reactants (E) of the reaction with CHD showing the orbital transformations of the newly formed O–H bond. All hydrogens apart from H_{CHD} and H_{OAu} of the reactant have been omitted for clarity. Calculated with PBE0-D3(BJ)/def2-SVP/(c)PCM(DMF).

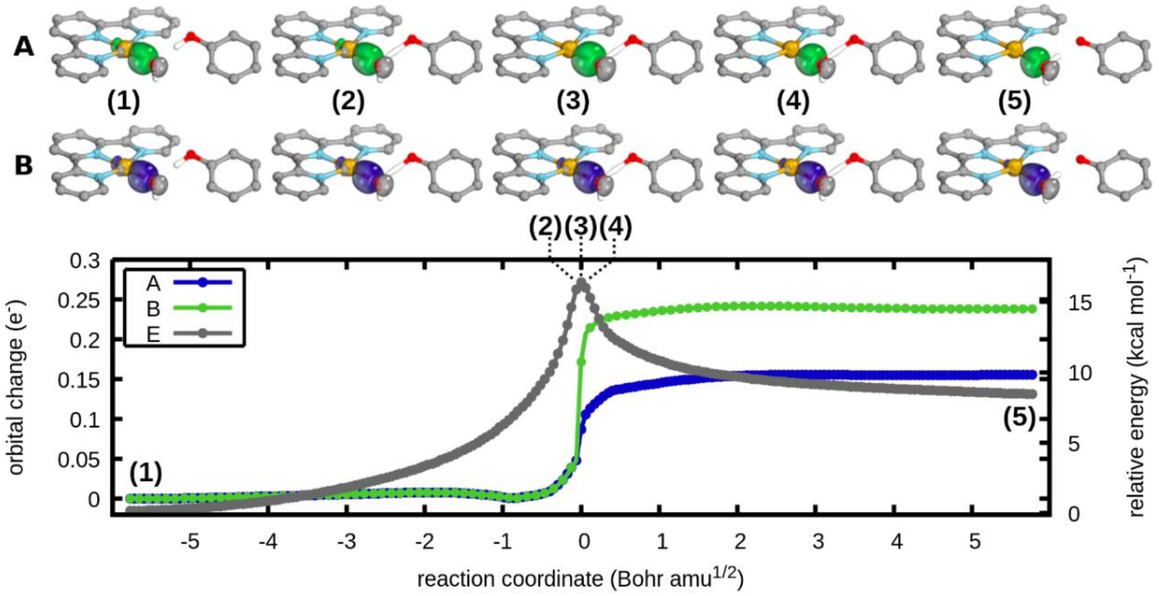

Figure S6: Electron flow analysis (A, and B) and relative energies reference to the fully relaxed separated reactants (E) of the reaction with PhOH showing the orbital transformations of relatively unaffected Au–O bond. All hydrogens apart from H_{OPh} and H_{OAu} of the reactant have been omitted for clarity. Calculated with PBE0-D3(BJ)/def2-SVP/(c)PCM(DMF).

5. Derived Curly Arrow Mechanisms

Although $[Au(OH)(terpy)]^{2+}$ cleaves both the C-H and O-H bonds of CHD and PhOH, respectively, via cPCET, their curly arrow mechanisms differ slightly (Scheme S1). In the case of CHD, the C-H σ-bond is cleaved homolytically, with one electron being transferred directly to the Au, as is expected from the definition of cPCET (Scheme 2). This cleavage is depicted in Figure 2. The new AuO-H bond is formed from a spin orbital of the AuOH lone pair (Figure S4, A (blue)) and a spin orbital from the Au-O bond (Figure S4, B, (red)). Thus, a slight deviation is observed in the orbital rearrangement compared to the definition outlined in Scheme 2. This slight difference does not however revoke the cPCET assignment as a proton and electron are still moving simultaneously but separately.

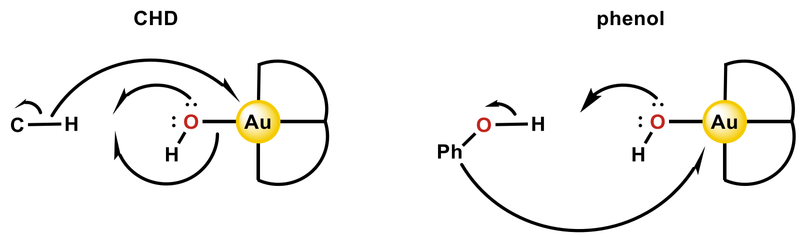

Scheme S1. Curly arrow mechanism for C-H bond cleavage of CHD (left) and O-H bond cleavage of PhOH (right) as derived from the IBO changes along the reaction coordinate for each reaction.

Despite the overall reaction indicating homolytic O-H bond cleavage, the PhOH O-H σ-bond is actually cleaved heterolytically. This can be from Figure 3B (green) and C (red), showing the O-H σ-bond transforming into the new PhO lone pair. The new AuO-H bond is formed from the AuO lone pair (Figure S7). Thus, a classical proton transfer is observed. This is coupled with simultaneous electron transfer from the π-system of PhOH (Figure 3A). Hence, the reaction with PhOH shows a true concerted proton coupled electron transfer reaction.

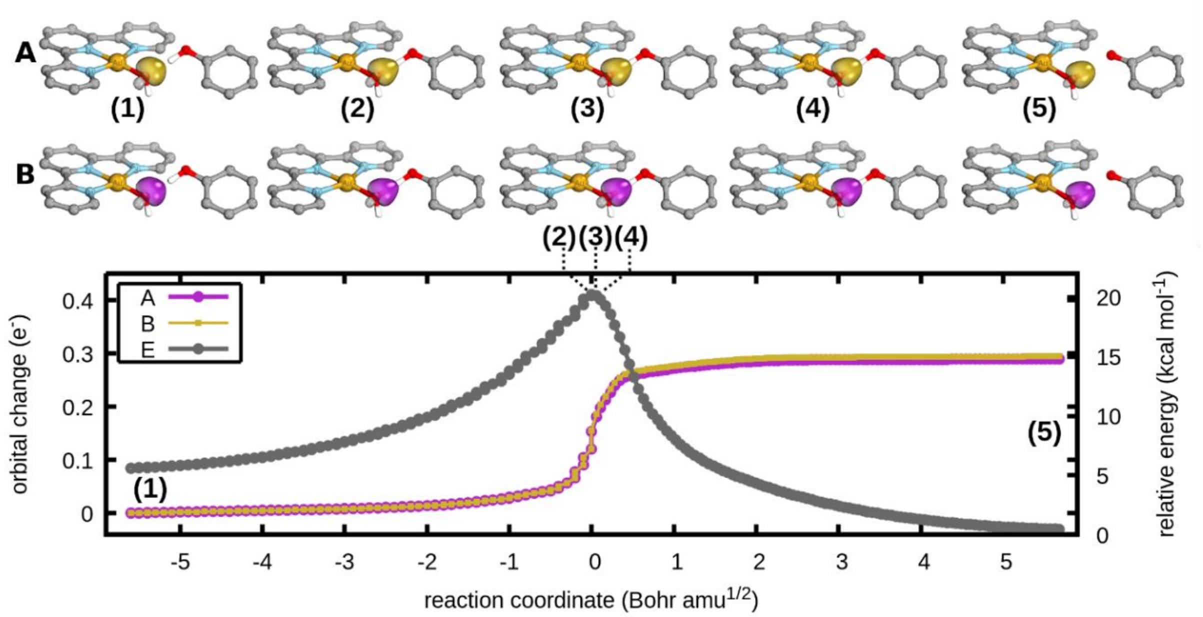

Figure S7. Electron flow analysis (A and B) and relative energies reference to the fully relaxed separated reactants (E) of the reaction with PhOH showing the orbital transformations of the AuOH lone pair. All hydrogens apart from H_{OPh} and H_{OAu} of the reactant have been omitted for clarity. Calculated with PBE0-D3(BJ)/def2-SVP/(c)PCM(DMF).

6. Cartesian Coordinates

Table S3. Cartesian coordinates (Å) of optimized structures

Au	0.802763000	0.004221000	0.000000000	$[(terpy)Au(OH)]^{2+}$ (C_s)
O	2.756485000	-0.072376000	0.000000000	
N	0.495247000	0.000884000	2.003761000	
N	-1.164173000	-0.008869000	0.000000000	$E_{PBE0-D3(BJ)/def2-SVP/PCM(DMF)}$ = -952.234816433
N	0.495247000	0.000884000	-2.003761000	
C	-1.771900000	0.000211000	1.194807000	Thermal correction to Gibbs Free Energy (298 K)$_{PBE0-D3(BJ)/def2-SVP/PCM(DMF)}$ = 0.204828
C	-3.163934000	0.009957000	1.219163000	
C	-3.844619000	0.013503000	0.000000000	
C	-3.163934000	0.009957000	-1.219163000	
C	-1.771900000	0.000211000	-1.194807000	
C	-0.831140000	-0.000921000	-2.332808000	
C	-1.203127000	-0.007726000	-3.667585000	
C	-0.211536000	-0.015515000	-4.650705000	
C	1.128022000	-0.018711000	-4.282027000	
C	1.452667000	-0.011142000	-2.928840000	
H	-3.711833000	0.015928000	2.161177000	
H	-4.935704000	0.021174000	0.000000000	
H	-3.711833000	0.015928000	-2.161177000	
H	-2.257729000	-0.009321000	-3.941629000	
H	-0.494604000	-0.021227000	-5.704623000	
C	-0.831140000	-0.000921000	2.332808000	
C	-1.203127000	-0.007726000	3.667585000	
H	-2.257729000	-0.009321000	3.941629000	
C	-0.211536000	-0.015515000	4.650705000	
H	-0.494604000	-0.021227000	5.704623000	
C	1.452667000	-0.011142000	2.928840000	
H	2.478670000	-0.018567000	2.553875000	
C	1.128022000	-0.018711000	4.282027000	
H	1.926102000	-0.027725000	5.024665000	
H	1.926102000	-0.027725000	-5.024665000	
H	2.478670000	-0.018567000	-2.553875000	
H	3.079183000	0.841093000	0.000000000	
Au	0.000000000	0.905937000	0.000000000	$[(terpy)Au(OH_2)]^{2+}$ (C_s)
O	-0.303560000	3.120942000	0.000000000	
N	2.133352000	0.439159000	0.000000000	
N	0.022358000	-1.183311000	0.000000000	$E_{PBE0-D3(BJ)/def2-SVP/PCM(DMF)}$ = -952.860935744
N	-2.119237000	0.404918000	0.000000000	

C	1.209638000	-1.791849000	0.000000000	Thermal correction to Gibbs Free Energy (298 K)$_{PBE0-D3(BJ)/def2-SVP/PCM(DMF)}$ = 0.210476
C	1.248990000	-3.186369000	0.000000000	
C	0.042035000	-3.882514000	0.000000000	
C	-1.175033000	-3.203865000	0.000000000	
C	-1.157380000	-1.809056000	0.000000000	
C	-2.349597000	-0.926954000	0.000000000	
C	-3.653428000	-1.412582000	0.000000000	
C	-4.714173000	-0.508493000	0.000000000	
C	-4.450683000	0.856512000	0.000000000	
C	-3.123600000	1.277404000	0.000000000	
H	2.195794000	-3.724954000	0.000000000	
H	0.049943000	-4.973619000	0.000000000	
H	-2.113843000	-3.756262000	0.000000000	
H	-3.845569000	-2.485010000	0.000000000	
H	-5.741788000	-0.876147000	0.000000000	
C	2.385808000	-0.889455000	0.000000000	
C	3.695894000	-1.358188000	0.000000000	
H	3.900488000	-2.428194000	0.000000000	
C	4.745960000	-0.442532000	0.000000000	
H	5.778189000	-0.796923000	0.000000000	
C	3.131190000	1.320904000	0.000000000	
H	2.856202000	2.377242000	0.000000000	
C	4.463465000	0.918290000	0.000000000	
H	5.255166000	1.668132000	0.000000000	
H	-5.253510000	1.594445000	0.000000000	
H	-2.848773000	2.334842000	0.000000000	
H	0.073175000	3.568511000	0.772637000	
H	0.073175000	3.568511000	-0.772637000	
C	-0.668415000	1.248951000	0.000000000	CHD (D_{2h})
C	-1.495664000	0.000000000	0.000000000	
C	-0.668415000	-1.248951000	0.000000000	E$_{PBE0-D3(BJ)/def2-SVP/PCM(DMF)}$ = -232.981225316
C	0.668415000	-1.248951000	0.000000000	
C	1.495664000	0.000000000	0.000000000	Thermal correction to Gibbs Free Energy (298 K)$_{PBE0-D3(BJ)/def2-SVP/PCM(DMF)}$ = 0.095266
C	0.668415000	1.248951000	0.000000000	
H	-1.206610000	2.202863000	0.000000000	
H	-1.206610000	-2.202863000	0.000000000	
H	1.206610000	-2.202863000	0.000000000	
H	1.206610000	2.202863000	0.000000000	
H	-2.178832000	0.000000000	0.871335000	
H	-2.178832000	0.000000000	-0.871335000	
H	2.178832000	0.000000000	-0.871335000	
H	2.178832000	0.000000000	0.871335000	
C	0.000058000	-0.626432000	1.250576000	[CHD-H]$^{\bullet}$ (C_s)
C	-0.000130000	-1.444081000	0.000000000	
C	0.000058000	-0.626432000	-1.250576000	E$_{PBE0-D3(BJ)/def2-SVP/PCM(DMF)}$ = -232.358887280
C	0.000058000	0.741076000	-1.224683000	
C	-0.000077000	1.456957000	0.000000000	Thermal correction to Gibbs Free Energy (298 K)$_{PBE0-D3(BJ)/def2-SVP/PCM(DMF)}$ = 0.080376
C	0.000058000	0.741076000	1.224683000	
H	0.000231000	-1.156308000	2.207467000	
H	0.000231000	-1.156308000	-2.207467000	
H	0.000235000	1.296472000	-2.167065000	
H	0.000235000	1.296472000	2.167065000	
H	0.866067000	-2.141098000	0.000000000	
H	-0.866471000	-2.140911000	0.000000000	
H	-0.000676000	2.548697000	0.000000000	
C	1.216427000	0.254448000	0.000000000	PhOH (C_s)
C	1.221313000	-1.137709000	0.000000000	
C	0.023502000	-1.855924000	0.000000000	E$_{PBE0-D3(BJ)/def2-SVP/PCM(DMF)}$ = -306.911004457
C	-1.185496000	-1.160314000	0.000000000	
C	-1.203686000	0.233705000	0.000000000	Thermal correction to Gibbs Free Energy (298 K)$_{PBE0-D3(BJ)/def2-SVP/PCM(DMF)}$ = 0.076718
C	0.000000000	0.948608000	0.000000000	
H	2.147981000	0.824569000	0.000000000	
H	2.176899000	-1.668373000	0.000000000	
H	0.033136000	-2.947878000	0.000000000	
H	-2.132567000	-1.705841000	0.000000000	
H	-2.154334000	0.774999000	0.000000000	
O	0.043802000	2.296771000	0.000000000	
H	-0.853883000	2.651465000	0.000000000	
C	0.000000000	1.237988000	0.291089000	[PhO]$^{\bullet}$ (C_{2V})
C	0.000000000	1.224804000	-1.085332000	
C	0.000000000	0.000000000	-1.782639000	E$_{PBE0-D3(BJ)/def2-SVP/PCM(DMF)}$ = -306.265591082
C	0.000000000	-1.224804000	-1.085332000	
C	0.000000000	-1.237988000	0.291089000	
C	0.000000000	0.000000000	1.048713000	Thermal correction to Gibbs Free Energy (298 K)$_{PBE0-D3(BJ)/def2-SVP/PCM(DMF)}$ = 0.063414
H	0.000000000	2.171034000	0.859387000	

H 0.000000000 2.162962000 -1.645138000<br>H 0.000000000 0.000000000 -2.874908000<br>H 0.000000000 -2.162962000 -1.645138000<br>H 0.000000000 -2.171034000 0.859387000<br>O 0.000000000 0.000000000 2.297610000	
Au -0.130526000 -0.799112000 -0.604605000<br>O -0.102799000 -2.751468000 -0.502010000<br>N -2.127856000 -0.501776000 -0.414760000<br>N -0.138905000 1.166437000 -0.595620000<br>N 1.864652000 -0.487092000 -0.754604000<br>C -1.332441000 1.768723000 -0.493577000<br>C -1.360920000 3.160792000 -0.489890000<br>C -0.147676000 3.845874000 -0.586334000<br>C 1.070558000 3.169557000 -0.679113000<br>C 1.051311000 1.777134000 -0.682301000<br>C 2.189336000 0.839846000 -0.754835000<br>C 3.521382000 1.216737000 -0.802797000<br>C 4.507289000 0.228724000 -0.837404000<br>C 4.143441000 -1.112199000 -0.824156000<br>C 2.792017000 -1.441524000 -0.781191000<br>H -2.301435000 3.705217000 -0.410395000<br>H -0.151513000 4.936918000 -0.583947000<br>H 2.008179000 3.720806000 -0.745719000<br>H 5.559739000 0.515304000 -0.870105000<br>C -2.460064000 0.822995000 -0.376332000<br>C -3.786151000 1.189229000 -0.211145000<br>H -4.062993000 2.242722000 -0.176949000<br>C -4.756363000 0.193057000 -0.081588000<br>H -5.803052000 0.471785000 0.051128000<br>C -3.039007000 -1.463228000 -0.287007000<br>H -2.660628000 -2.487588000 -0.315835000<br>C -4.383222000 -1.144684000 -0.117355000<br>H -5.114954000 -1.946070000 -0.013519000<br>H 4.888384000 -1.907832000 -0.844373000<br>H 2.422241000 -2.469179000 -0.760457000<br>H -0.158028000 -3.091726000 -1.407493000<br>H 3.791347000 2.272330000 -0.804384000<br>C 0.255833000 1.435104000 2.798591000<br>C 0.999776000 -1.454036000 2.725628000<br>C -0.417731000 -0.968385000 2.713428000<br>C -0.751839000 0.328537000 2.742319000<br>H 0.162142000 1.974033000 3.761775000<br>H 1.148661000 -2.207683000 1.929522000<br>H 1.190479000 -2.021022000 3.657786000<br>H 0.018224000 2.214410000 2.050733000<br>C 1.996969000 -0.343547000 2.595615000<br>H 3.048677000 -0.626141000 2.480663000<br>C 1.663171000 0.952042000 2.623618000<br>H 2.445367000 1.712830000 2.533129000<br>H -1.206435000 -1.727494000 2.691052000<br>H -1.809719000 0.612522000 2.747097000	RC_{CHD}<br><br>$E_{PBE0-D3(BJ)/def2-SVP/PCM(DMF)}$ = -1185.23391278<br><br>Thermal correction to Gibbs Free Energy (298 K)$_{PBE0-D3(BJ)/def2-SVP/PCM(DMF)}$ = 0.318247<br><br>$E_{PBE-D3(BJ)/def2-SVP/cPCM(DMF)}$ = -1185.227858295741<br><br>$E_{PBE100-D3(BJ)/def2-SVP/cPCM(DMF)}$ = -1185.494404791345
Au -0.274201000 -0.302770000 -0.365292000<br>O 1.200563000 -1.657197000 -0.814072000<br>N -2.000271000 -1.502651000 -0.141469000<br>N -1.660440000 1.099705000 0.175247000<br>N 0.895908000 1.469781000 -0.395320000<br>C -2.911263000 0.675233000 0.372791000<br>C -3.891310000 1.617261000 0.682173000<br>C -3.514514000 2.957819000 0.765606000<br>C -2.196047000 3.353755000 0.540133000<br>C -1.255942000 2.371467000 0.231364000<br>C 0.183404000 2.575270000 -0.059324000<br>C 0.812547000 3.813133000 -0.003313000<br>C 2.173962000 3.898422000 -0.294229000<br>C 2.877383000 2.747839000 -0.630849000<br>C 2.193632000 1.535560000 -0.669089000<br>H -4.926705000 1.322955000 0.850994000<br>H -4.266239000 3.711414000 1.005611000<br>H -1.917117000 4.405270000 0.600145000<br>H 2.678297000 4.865412000 -0.253351000<br>C -3.095179000 -0.788287000 0.226672000<br>C -4.304409000 -1.435931000 0.448310000<br>H -5.185777000 -0.868875000 0.746889000<br>C -4.374857000 -2.819430000 0.289219000<br>H -5.319298000 -3.338734000 0.460788000	TS_{CHD}<br><br>$E_{PBE0-D3(BJ)/def2-SVP/PCM(DMF)}$ = -1185.20164262<br><br>Thermal correction to Gibbs Free Energy (298 K)$_{PBE0-D3(BJ)/def2-SVP/PCM(DMF)}$ = 0.310451<br><br>$\langle S^2 \rangle$ = 0.4191<br><br>(S = 1) $E_{PBE0-D3(BJ)/def2-SVP/PCM(DMF)}$ = -1185.18606872<br><br>$E_{PBE-D3(BJ)/def2-SVP/cPCM(DMF)}$ = -1185.210013676305<br><br>$E_{PBE100-D3(BJ)/def2-SVP/cPCM(DMF)}$ = -1185.453093113966<br><br>$E_{CASSCF(6,6)/def2-SVP}$ = -1178.652106919219

C	-2.053785000	-2.824632000	-0.290258000
H	-1.124153000	-3.319844000	-0.578128000
C	-3.237647000	-3.526331000	-0.083358000
H	-3.253559000	-4.608529000	-0.215157000
H	3.942633000	2.775088000	-0.863185000
H	2.692710000	0.591749000	-0.914237000
H	1.120003000	-1.928458000	-1.743188000
H	0.249153000	4.705747000	0.267829000
C	4.695107000	-0.282736000	1.393005000
C	3.529034000	-2.536551000	-0.062625000
C	3.409396000	-2.416882000	1.390863000
C	3.915542000	-1.361531000	2.063723000
H	5.653829000	-0.119952000	1.924425000
H	2.388749000	-2.169064000	-0.439986000
H	3.542513000	-3.561634000	-0.460123000
H	4.178788000	0.692215000	1.525649000
C	4.435495000	-1.590634000	-0.712423000
H	4.663199000	-1.736356000	-1.772206000
C	4.952376000	-0.530313000	-0.054340000
H	5.605831000	0.172302000	-0.580180000
H	2.852674000	-3.189757000	1.928232000
H	3.777880000	-1.287405000	3.146303000

IC_{CHD}

$E_{PBE0\text{-}D3(BJ)/def2\text{-}SVP/PCM(DMF)}$ = -1185.23807514

Thermal correction to Gibbs Free Energy (298 K)$_{PBE0\text{-}D3(BJ)/def2\text{-}SVP/PCM(DMF)}$ = 0.313942

$\langle S^2 \rangle$ = 1.0468

(*S* = 1) $E_{PBE0\text{-}D3(BJ)/def2\text{-}SVP/PCM(DMF)}$ = -1185.23799011

$E_{PBE\text{-}D3(BJ)/def2\text{-}SVP/cPCM(DMF)}$ = -1185.261688888865

$E_{PBE100\text{-}D3(BJ)/def2\text{-}SVP/cPCM(DMF)}$ = -1185.529433599897

Au	-0.107800000	-0.313786000	-0.695645000
O	0.199913000	-2.338526000	-1.533567000
N	-2.260057000	-0.496630000	-0.370597000
N	-0.609622000	1.539875000	0.121381000
N	1.812003000	0.741097000	-0.663896000
C	-1.890284000	1.744434000	0.442492000
C	-2.257169000	2.978428000	0.979332000
C	-1.269954000	3.945956000	1.154689000
C	0.053614000	3.687558000	0.804709000
C	0.367710000	2.436016000	0.274405000
C	1.717914000	1.986193000	-0.144293000
C	2.852577000	2.780840000	-0.010452000
C	4.086407000	2.278221000	-0.418863000
C	4.159095000	0.996442000	-0.950901000
C	2.987109000	0.252690000	-1.055429000
H	-3.289655000	3.188456000	1.255583000
H	-1.536869000	4.918549000	1.571162000
H	0.818071000	4.450768000	0.943698000
H	4.984161000	2.890492000	-0.317785000
C	-2.806221000	0.605529000	0.187215000
C	-4.162669000	0.633758000	0.497987000
H	-4.611409000	1.517558000	0.950332000
C	-4.943223000	-0.488818000	0.227103000
H	-6.007803000	-0.481368000	0.467539000
C	-2.994199000	-1.574034000	-0.633482000
H	-2.470774000	-2.421660000	-1.082297000
C	-4.355939000	-1.610840000	-0.346710000
H	-4.934278000	-2.507306000	-0.572365000
H	5.104761000	0.565744000	-1.281407000
H	2.990405000	-0.761918000	-1.457291000
H	0.731529000	-2.385170000	-2.341806000
H	2.781947000	3.781991000	0.413128000
C	2.010371000	-1.182237000	2.306847000
C	1.246396000	-3.638321000	0.977358000
C	0.277965000	-2.879944000	1.692016000
C	0.611744000	-1.701559000	2.302932000
H	2.386796000	-1.138464000	3.353467000
H	0.652212000	-2.897310000	-0.866286000
H	0.978979000	-4.608076000	0.550025000
H	2.031066000	-0.114300000	2.009541000
C	2.576127000	-3.149204000	0.868688000
H	3.308761000	-3.720431000	0.292331000
C	2.952953000	-1.980286000	1.471758000
H	3.982159000	-1.623416000	1.379188000
H	-0.752618000	-3.241563000	1.742803000
H	-0.148760000	-1.130602000	2.842335000

RC_{PhOH}

$E_{PBE0\text{-}D3(BJ)/def2\text{-}SVP/PCM(DMF)}$ = -1259.16450811

Thermal correction to Gibbs Free Energy (298 K)$_{PBE0\text{-}D3(BJ)/def2\text{-}SVP/PCM(DMF)}$ = 0.300795

Au	-0.522851000	-0.144808000	-0.234429000
O	1.296123000	-0.823112000	-0.581275000
N	-0.203680000	1.867027000	-0.249514000
N	-2.350549000	0.447953000	0.151148000
N	-1.405423000	-1.962223000	-0.084027000
C	-2.568192000	1.770717000	0.196453000
C	-3.866792000	2.209898000	0.439958000

C	-4.866537000	1.252722000	0.619501000
C	-4.588737000	-0.113931000	0.556889000
C	-3.277879000	-0.509984000	0.310556000
C	-2.741261000	-1.877855000	0.188783000
C	-3.482450000	-3.039511000	0.333401000
C	-2.844025000	-4.274291000	0.199224000
C	-1.482849000	-4.323770000	-0.074750000
C	-0.780360000	-3.130250000	-0.212291000
H	-4.100781000	3.273050000	0.487752000
H	-5.889655000	1.580409000	0.810300000
H	-5.380217000	-0.849839000	0.694910000
H	-4.548942000	-2.985705000	0.551699000
H	-3.418989000	-5.194782000	0.313060000
C	-1.353228000	2.575393000	-0.027475000
C	-1.316659000	3.960276000	-0.015619000
H	-2.228971000	4.529469000	0.160907000
C	-0.100832000	4.612716000	-0.227353000
H	-0.062383000	5.703220000	-0.220979000
C	0.965796000	2.477105000	-0.440294000
H	1.843806000	1.841348000	-0.570961000
C	1.050957000	3.866618000	-0.436591000
H	2.020232000	4.339833000	-0.594861000
H	-0.954489000	-5.271212000	-0.182734000
H	0.290567000	-3.078964000	-0.422197000
H	1.450072000	-0.804190000	-1.538407000
O	3.242805000	0.473594000	0.625157000
H	2.550197000	-0.107462000	0.220156000
C	4.466952000	-0.050883000	0.446167000
C	5.578103000	0.682687000	0.884981000
C	6.862484000	0.171805000	0.719370000
C	7.063607000	-1.072051000	0.116569000
C	5.956721000	-1.801590000	-0.318284000
C	4.665540000	-1.301580000	-0.157322000
H	5.411742000	1.655196000	1.353943000
H	7.719078000	0.755658000	1.066366000
H	8.073069000	-1.468553000	-0.011408000
H	6.095349000	-2.777910000	-0.790087000
H	3.799498000	-1.878333000	-0.493135000

$E_{PBE-D3(BJ)/def2-SVP/cPCM(DMF)}$ = -1259.171582243415

$E_{PBE100-D3(BJ)/def2-SVP/cPCM(DMF)}$ = -1259.313890301627

TS$_{PhOH}$

$E_{PBE0-D3(BJ)/def2-SVP/PCM(DMF)}$ = -1259.13840335

Thermal correction to Gibbs Free Energy (298 K)$_{PBE0-D3(BJ)/def2-SVP/PCM(DMF)}$ = 0.292785

$\langle S^2 \rangle$ = 0.6291

(S = 1) $E_{PBE0-D3(BJ)/def2-SVP/PCM(DMF)}$ = -1259.13662477

$E_{PBE-D3(BJ)/def2-SVP/cPCM(DMF)}$ = -1259.159830782162

$E_{PBE100-D3(BJ)/def2-SVP/cPCM(DMF)}$ = -1259.385555949358

$E_{CASSCF(12,12)/def2-SVP}$ = -1252.423578347054

Au	-0.443287000	-0.218993000	-0.138226000
O	1.342911000	-1.260978000	-0.321488000
N	0.053342000	1.874399000	-0.142774000
N	-2.277944000	0.637724000	0.053420000
N	-1.699525000	-1.943524000	-0.013672000
C	-2.346969000	1.973892000	0.055747000
C	-3.597532000	2.578660000	0.173905000
C	-4.722228000	1.762902000	0.278912000
C	-4.603972000	0.374160000	0.267184000
C	-3.332367000	-0.183750000	0.150701000
C	-3.009252000	-1.628239000	0.120547000
C	-3.956781000	-2.640315000	0.223643000
C	-3.535178000	-3.969353000	0.185665000
C	-2.182838000	-4.260558000	0.047652000
C	-1.281116000	-3.204051000	-0.049632000
H	-3.699586000	3.663032000	0.184805000
H	-5.709751000	2.217631000	0.370717000
H	-5.489122000	-0.255672000	0.346773000
H	-5.014413000	-2.402461000	0.333816000
H	-4.269463000	-4.772840000	0.265614000
C	-1.045105000	2.665765000	-0.067042000
C	-0.910665000	4.048771000	-0.097326000
H	-1.789133000	4.690778000	-0.039567000
C	0.363444000	4.605988000	-0.200729000
H	0.481731000	5.690571000	-0.227031000
C	1.273439000	2.392711000	-0.229786000
H	2.109815000	1.686837000	-0.246237000
C	1.471981000	3.771024000	-0.262230000
H	2.485059000	4.168008000	-0.332815000
H	-1.818818000	-5.287894000	0.015680000
H	-0.203210000	-3.351050000	-0.155332000
H	1.550157000	-1.353322000	-1.263373000
O	3.108762000	-0.015057000	0.694096000
H	2.266730000	-0.675793000	0.204094000
C	4.322838000	-0.371750000	0.432879000
C	5.397700000	0.472649000	0.844987000
C	6.701141000	0.128855000	0.552118000

C 6.983119000 -1.060559000 -0.142801000<br>C 5.937633000 -1.911076000 -0.539003000<br>C 4.625399000 -1.585409000 -0.257990000<br>H 5.149368000 1.392761000 1.377467000<br>H 7.519313000 0.783303000 0.860283000<br>H 8.017603000 -1.329054000 -0.365871000<br>H 6.164881000 -2.842090000 -1.062644000<br>H 3.807842000 -2.256122000 -0.530586000	
Au -0.408003000 -0.212532000 -0.481121000<br>O 1.178735000 -1.445844000 -1.322511000<br>N 0.227757000 1.904143000 -0.357089000<br>N -2.082182000 0.750314000 0.318076000<br>N -1.824123000 -1.861735000 -0.174704000<br>C -2.025061000 2.072784000 0.493913000<br>C -3.136672000 2.734320000 1.017155000<br>C -4.266686000 1.985438000 1.335057000<br>C -4.285103000 0.607140000 1.134589000<br>C -3.147265000 -0.004513000 0.608907000<br>C -3.004226000 -1.455820000 0.338429000<br>C -4.012002000 -2.381480000 0.595281000<br>C -3.781502000 -3.727940000 0.319845000<br>C -2.554025000 -4.120680000 -0.202148000<br>C -1.590253000 -3.144046000 -0.437569000<br>H -3.127792000 3.811495000 1.177721000<br>H -5.146491000 2.483168000 1.745909000<br>H -5.171739000 0.026170000 1.384964000<br>H -4.968268000 -2.062073000 1.008149000<br>H -4.562337000 -4.464564000 0.517044000<br>C -0.746727000 2.716251000 0.107557000<br>C -0.533951000 4.087917000 0.213332000<br>H -1.320390000 4.744150000 0.584151000<br>C 0.699760000 4.614874000 -0.162244000<br>H 0.879851000 5.688627000 -0.086033000<br>C 1.412777000 2.397671000 -0.712599000<br>H 2.163715000 1.682144000 -1.058724000<br>C 1.690646000 3.759818000 -0.628830000<br>H 2.672035000 4.129832000 -0.927747000<br>H -2.335500000 -5.165171000 -0.426794000<br>H -0.604726000 -3.385986000 -0.842536000<br>H 1.056577000 -1.571420000 -2.274907000<br>O 3.360296000 -0.175827000 -0.957363000<br>H 2.081330000 -0.995860000 -1.211656000<br>C 4.230220000 -0.567371000 -0.135805000<br>C 5.371769000 0.266918000 0.168114000<br>C 6.320600000 -0.157931000 1.066469000<br>C 6.187431000 -1.412929000 1.697282000<br>C 5.085359000 -2.249804000 1.419740000<br>C 4.121651000 -1.849755000 0.526242000<br>H 5.441856000 1.231227000 -0.339143000<br>H 7.181985000 0.472275000 1.296762000<br>H 6.947269000 -1.741609000 2.409412000<br>H 5.003775000 -3.217075000 1.919619000<br>H 3.258281000 -2.476149000 0.291098000	IC_{PhOH}<br><br>$E_{PBE0\text{-}D3(BJ)/def2\text{-}SVP/PCM(DMF)}$ = -1259.15295664<br><br>Thermal correction to Gibbs Free Energy (298 K)$_{PBE0\text{-}D3(BJ)/def2\text{-}SVP/PCM(DMF)}$ = 0.295378<br><br>$\langle S^2 \rangle$ = 1.0433<br><br>(S = 1) $E_{PBE0\text{-}D3(BJ)/def2\text{-}SVP/PCM(DMF)}$ = -1259.15289314<br><br>$E_{PBE\text{-}D3(BJ)/def2\text{-}SVP/cPCM(DMF)}$ = -1259.154576831353<br><br>$E_{PBE100\text{-}D3(BJ)/def2\text{-}SVP/cPCM(DMF)}$ = -1259.407346038412
Coordinates obtained from ref. [10h]	$TS_{(N\text{^}N\text{^}N)Cu\text{-}OH\text{-}CHD}$<br><br>$E_{PBE0\text{-}D3(BJ)/def2\text{-}SVP/cPCM(DMF)}$ = -3464.364941110348<br><br>$\langle S^2 \rangle$ = 0.7154
Coordinates obtained from ref. [10h]	$TS_{(N\text{^}N\text{^}N)Cu\text{-}OH\text{-}DHA}$<br><br>$E_{PBE0\text{-}D3(BJ)/def2\text{-}SVP/cPCM(DMF)}$ = -3771.139851589211<br><br>$\langle S^2 \rangle$ = 0.6992

7. Intrinsic Bond Orbital Energy Changes

Table S4: Reaction coordinate, energies relative to the separated reactants (from the Gaussian IRC), and IBO changes (|$q_i(s)$-$q_i(0)$|, from IboView) for the reaction with CHD in DMF.

**reaction coordinate**	**relative energy**	**IBO change (e^-)**				
		**Figure S3**			**Figure 2**	
**(bohr amu$^{1/2}$)**	**(kcal mol^{-1})**	**B**	**C**	**A**	**A**	**B**
-5.68	5.59	0.0000	0.0000	0.0000	0.0000	0.0000
-5.63	5.60	0.0003	0.0003	0.0005	0.0001	0.0001
-5.57	5.62	0.0005	0.0005	0.0005	0.0002	0.0002
-5.51	5.64	0.0008	0.0008	0.0005	0.0004	0.0004
-5.46	5.65	0.0010	0.0010	0.0006	0.0004	0.0004
-5.40	5.67	0.0013	0.0013	0.0007	0.0006	0.0006
-5.34	5.69	0.0015	0.0015	0.0007	0.0007	0.0006
-5.29	5.71	0.0018	0.0018	0.0007	0.0009	0.0009
-5.23	5.74	0.0020	0.0020	0.0008	0.0009	0.0009
-5.17	5.76	0.0023	0.0023	0.0009	0.0011	0.0011
-5.12	5.78	0.0026	0.0026	0.0010	0.0012	0.0012
-5.06	5.81	0.0029	0.0029	0.0011	0.0014	0.0014
-5.00	5.84	0.0031	0.0031	0.0012	0.0016	0.0016
-4.95	5.87	0.0034	0.0034	0.0013	0.0017	0.0017
-4.89	5.90	0.0037	0.0037	0.0014	0.0019	0.0019
-4.83	5.93	0.0040	0.0040	0.0015	0.0020	0.0020
-4.78	5.97	0.0042	0.0042	0.0016	0.0022	0.0022
-4.72	6.00	0.0045	0.0045	0.0017	0.0023	0.0023
-4.66	6.04	0.0048	0.0048	0.0018	0.0025	0.0025
-4.61	6.08	0.0050	0.0051	0.0019	0.0027	0.0027
-4.55	6.12	0.0053	0.0053	0.0020	0.0029	0.0029
-4.49	6.16	0.0056	0.0056	0.0022	0.0030	0.0030
-4.43	6.20	0.0058	0.0059	0.0023	0.0032	0.0032
-4.38	6.24	0.0061	0.0061	0.0024	0.0034	0.0034
-4.32	6.29	0.0064	0.0064	0.0025	0.0036	0.0036
-4.26	6.34	0.0066	0.0067	0.0027	0.0038	0.0038
-4.21	6.39	0.0069	0.0069	0.0028	0.0040	0.0040
-4.15	6.44	0.0072	0.0072	0.0030	0.0042	0.0042
-4.09	6.49	0.0075	0.0075	0.0031	0.0045	0.0045
-4.04	6.54	0.0077	0.0077	0.0033	0.0047	0.0047
-3.98	6.60	0.0080	0.0080	0.0034	0.0049	0.0049
-3.92	6.66	0.0083	0.0083	0.0036	0.0052	0.0052
-3.87	6.72	0.0085	0.0086	0.0037	0.0054	0.0054
-3.81	6.78	0.0088	0.0088	0.0039	0.0057	0.0057
-3.75	6.84	0.0091	0.0091	0.0041	0.0059	0.0059
-3.70	6.91	0.0094	0.0094	0.0043	0.0062	0.0062
-3.64	6.97	0.0097	0.0097	0.0044	0.0065	0.0065
-3.58	7.04	0.0099	0.0099	0.0046	0.0068	0.0068
-3.53	7.11	0.0102	0.0103	0.0048	0.0071	0.0071
-3.47	7.18	0.0105	0.0105	0.0050	0.0074	0.0074
-3.41	7.26	0.0108	0.0108	0.0052	0.0077	0.0077
-3.35	7.34	0.0111	0.0111	0.0055	0.0080	0.0080
-3.30	7.42	0.0114	0.0114	0.0057	0.0083	0.0083
-3.24	7.50	0.0117	0.0117	0.0059	0.0087	0.0087
-3.18	7.58	0.0120	0.0120	0.0062	0.0090	0.0090
-3.13	7.67	0.0123	0.0124	0.0064	0.0094	0.0094

-3.07	7.76	0.0126	0.0127	0.0067	0.0098	0.0098
-3.01	7.85	0.0130	0.0130	0.0069	0.0102	0.0102
-2.96	7.94	0.0133	0.0133	0.0072	0.0106	0.0106
-2.90	8.04	0.0136	0.0136	0.0075	0.0110	0.0110
-2.84	8.14	0.0140	0.0139	0.0078	0.0114	0.0114
-2.79	8.24	0.0143	0.0143	0.0081	0.0119	0.0119
-2.73	8.35	0.0146	0.0146	0.0084	0.0124	0.0124
-2.67	8.46	0.0150	0.0150	0.0087	0.0129	0.0129
-2.61	8.57	0.0153	0.0153	0.0091	0.0134	0.0134
-2.56	8.69	0.0157	0.0156	0.0095	0.0139	0.0139
-2.50	8.81	0.0160	0.0159	0.0098	0.0145	0.0145
-2.44	8.93	0.0164	0.0163	0.0102	0.0150	0.0150
-2.39	9.06	0.0167	0.0166	0.0106	0.0156	0.0156
-2.33	9.19	0.0171	0.0170	0.0111	0.0163	0.0163
-2.27	9.32	0.0174	0.0174	0.0115	0.0169	0.0169
-2.22	9.46	0.0178	0.0177	0.0120	0.0176	0.0176
-2.16	9.60	0.0181	0.0181	0.0125	0.0183	0.0183
-2.10	9.75	0.0185	0.0184	0.0130	0.0191	0.0191
-2.05	9.90	0.0189	0.0188	0.0135	0.0199	0.0199
-1.99	10.06	0.0192	0.0191	0.0140	0.0207	0.0207
-1.93	10.23	0.0195	0.0195	0.0146	0.0215	0.0215
-1.88	10.39	0.0199	0.0199	0.0152	0.0224	0.0224
-1.82	10.57	0.0203	0.0202	0.0158	0.0234	0.0234
-1.76	10.75	0.0206	0.0206	0.0165	0.0244	0.0244
-1.71	10.93	0.0209	0.0208	0.0171	0.0255	0.0255
-1.65	11.12	0.0212	0.0211	0.0179	0.0266	0.0266
-1.59	11.32	0.0215	0.0214	0.0187	0.0278	0.0278
-1.54	11.53	0.0217	0.0217	0.0194	0.0290	0.0290
-1.48	11.74	0.0220	0.0221	0.0202	0.0303	0.0303
-1.42	11.97	0.0223	0.0224	0.0211	0.0316	0.0316
-1.36	12.20	0.0226	0.0226	0.0219	0.0331	0.0331
-1.31	12.44	0.0229	0.0229	0.0228	0.0345	0.0345
-1.25	12.69	0.0233	0.0232	0.0237	0.0361	0.0361
-1.19	12.95	0.0235	0.0235	0.0247	0.0377	0.0377
-1.14	13.21	0.0238	0.0238	0.0257	0.0394	0.0394
-1.08	13.49	0.0242	0.0241	0.0267	0.0412	0.0412
-1.02	13.78	0.0245	0.0245	0.0278	0.0431	0.0431
-0.97	14.07	0.0248	0.0248	0.0288	0.0451	0.0451
-0.91	14.38	0.0251	0.0252	0.0299	0.0471	0.0471
-0.85	14.70	0.0255	0.0255	0.0311	0.0493	0.0493
-0.80	15.03	0.0258	0.0259	0.0323	0.0515	0.0515
-0.74	15.36	0.0262	0.0262	0.0335	0.0539	0.0539
-0.68	15.71	0.0266	0.0266	0.0347	0.0564	0.0564
-0.63	16.07	0.0271	0.0271	0.0360	0.0591	0.0591
-0.57	16.44	0.0275	0.0275	0.0373	0.0619	0.0619
-0.51	16.81	0.0280	0.0280	0.0387	0.0649	0.0649
-0.45	17.20	0.0285	0.0285	0.0401	0.0681	0.0681
-0.40	17.59	0.0290	0.0290	0.0417	0.0717	0.0717
-0.34	18.00	0.0295	0.0295	0.0435	0.0758	0.0758
-0.28	18.42	0.0301	0.0301	0.0457	0.0812	0.0812
-0.23	18.88	0.0307	0.0307	0.0488	0.0886	0.0886
-0.17	19.39	0.0597	0.0274	0.0538	0.1118	0.0912
-0.11	19.85	0.3268	0.0762	0.0630	0.8161	0.0977

-0.06	20.14	0.3553	0.1015	0.0731	0.9588	0.1112
0.00	20.25	0.3529	0.1183	0.0847	1.0253	0.1264
0.06	20.15	0.3539	0.1306	0.0981	1.0712	0.1430
0.11	19.83	0.3571	0.1407	0.1144	1.1048	0.1608
0.17	19.30	0.3613	0.1508	0.1355	1.1299	0.1802
0.23	18.57	0.3658	0.1642	0.1641	1.1488	0.2012
0.28	17.68	0.3702	0.1825	0.1968	1.1630	0.2234
0.34	16.65	0.3742	0.1981	0.2248	1.1738	0.2455
0.40	15.54	0.3779	0.2081	0.2472	1.1820	0.2670
0.46	14.39	0.3811	0.2145	0.2665	1.1884	0.2876
0.51	13.28	0.3838	0.2189	0.2836	1.1933	0.3069
0.57	12.29	0.3861	0.2222	0.2985	1.1971	0.3244
0.63	11.45	0.3878	0.2245	0.3111	1.2000	0.3393
0.68	10.77	0.3890	0.2260	0.3206	1.2021	0.3506
0.74	10.19	0.3896	0.2267	0.3269	1.2035	0.3580
0.80	9.67	0.3899	0.2270	0.3311	1.2045	0.3627
0.85	9.19	0.3901	0.2271	0.3342	1.2056	0.3663
0.91	8.75	0.3902	0.2272	0.3370	1.2092	0.3693
0.97	8.34	0.3904	0.2272	0.3395	1.2067	0.3722
1.02	7.95	0.3905	0.2274	0.3419	1.2073	0.3750
1.08	7.60	0.3907	0.2275	0.3443	1.2105	0.3776
1.14	7.27	0.3909	0.2277	0.3465	1.2101	0.3802
1.19	6.97	0.3910	0.2279	0.3487	1.2090	0.3827
1.25	6.69	0.3912	0.2282	0.3507	1.2092	0.3851
1.31	6.44	0.3914	0.2285	0.3526	1.2095	0.3874
1.36	6.20	0.3916	0.2288	0.3545	1.2098	0.3895
1.42	5.98	0.3918	0.2291	0.3562	1.2100	0.3915
1.48	5.77	0.3919	0.2295	0.3578	1.2103	0.3934
1.54	5.58	0.3921	0.2298	0.3593	1.2111	0.3951
1.59	5.40	0.3923	0.2302	0.3607	1.2106	0.3967
1.65	5.23	0.3925	0.2306	0.3619	1.2108	0.3980
1.71	5.06	0.3926	0.2310	0.3632	1.2110	0.3995
1.76	4.91	0.3928	0.2314	0.3642	1.2111	0.4005
1.82	4.76	0.3929	0.2317	0.3652	1.2113	0.4016
1.88	4.61	0.3931	0.2320	0.3661	1.2115	0.4026
1.93	4.47	0.3932	0.2323	0.3670	1.2117	0.4035
1.99	4.33	0.3933	0.2326	0.3678	1.2118	0.4044
2.05	4.20	0.3934	0.2329	0.3685	1.2119	0.4052
2.10	4.07	0.3936	0.2331	0.3693	1.2120	0.4059
2.16	3.94	0.3937	0.2333	0.3700	1.2122	0.4067
2.22	3.82	0.3938	0.2335	0.3706	1.2123	0.4074
2.27	3.70	0.3939	0.2337	0.3713	1.2124	0.4081
2.33	3.58	0.3940	0.2338	0.3719	1.2124	0.4087
2.39	3.46	0.3941	0.2339	0.3725	1.2125	0.4094
2.44	3.35	0.3942	0.2340	0.3731	1.2125	0.4100
2.50	3.24	0.3943	0.2341	0.3737	1.2127	0.4106
2.56	3.14	0.3944	0.2342	0.3743	1.2127	0.4112
2.61	3.04	0.3945	0.2342	0.3749	1.2128	0.4119
2.67	2.94	0.3946	0.2343	0.3754	1.2129	0.4125
2.73	2.84	0.3947	0.2344	0.3760	1.2129	0.4131
2.79	2.74	0.3947	0.2344	0.3765	1.2131	0.4136
2.84	2.65	0.3948	0.2344	0.3770	1.2133	0.4142
2.90	2.56	0.3949	0.2345	0.3775	1.2133	0.4148

2.96	2.48	0.3950	0.2345	0.3780	1.2133	0.4153
3.01	2.39	0.3951	0.2345	0.3785	1.2131	0.4158
3.07	2.31	0.3951	0.2346	0.3789	1.2135	0.4164
3.13	2.23	0.3952	0.2346	0.3794	1.2135	0.4169
3.18	2.15	0.3953	0.2346	0.3798	1.2135	0.4174
3.24	2.08	0.3954	0.2347	0.3803	1.2135	0.4179
3.30	2.00	0.3954	0.2347	0.3807	1.2136	0.4184
3.35	1.93	0.3955	0.2348	0.3811	1.2137	0.4189
3.41	1.86	0.3956	0.2348	0.3816	1.2137	0.4193
3.47	1.80	0.3956	0.2348	0.3819	1.2137	0.4198
3.52	1.73	0.3957	0.2349	0.3823	1.2138	0.4203
3.58	1.67	0.3958	0.2350	0.3827	1.2138	0.4207
3.64	1.61	0.3958	0.2350	0.3831	1.2143	0.4212
3.70	1.55	0.3959	0.2351	0.3835	1.2143	0.4216
3.75	1.49	0.3960	0.2352	0.3838	1.2142	0.4220
3.81	1.44	0.3960	0.2352	0.3841	1.2143	0.4224
3.87	1.38	0.3961	0.2353	0.3845	1.2142	0.4228
3.92	1.33	0.3961	0.2354	0.3848	1.2143	0.4232
3.98	1.28	0.3962	0.2355	0.3851	1.2143	0.4236
4.04	1.24	0.3962	0.2356	0.3854	1.2143	0.4239
4.09	1.19	0.3963	0.2356	0.3857	1.2145	0.4243
4.15	1.14	0.3964	0.2357	0.3860	1.2143	0.4246
4.21	1.10	0.3964	0.2358	0.3863	1.2147	0.4250
4.26	1.06	0.3964	0.2359	0.3865	1.2158	0.4253
4.32	1.02	0.3965	0.2360	0.3868	1.2159	0.4256
4.38	0.98	0.3965	0.2361	0.3871	1.2159	0.4259
4.43	0.94	0.3966	0.2362	0.3873	1.2159	0.4262
4.49	0.91	0.3966	0.2363	0.3875	1.2160	0.4265
4.55	0.87	0.3967	0.2363	0.3878	1.2160	0.4268
4.60	0.84	0.3967	0.2364	0.3880	1.2160	0.4271
4.66	0.81	0.3967	0.2365	0.3882	1.2160	0.4274
4.72	0.78	0.3968	0.2365	0.3884	1.2160	0.4276
4.78	0.75	0.3968	0.2366	0.3886	1.2159	0.4279
4.83	0.72	0.3968	0.2366	0.3888	1.2161	0.4282
4.89	0.69	0.3969	0.2367	0.3890	1.2161	0.4284
4.95	0.67	0.3969	0.2367	0.3892	1.2160	0.4287
5.00	0.64	0.3969	0.2367	0.3894	1.2160	0.4289
5.06	0.62	0.3969	0.2367	0.3896	1.2160	0.4291
5.12	0.60	0.3970	0.2367	0.3897	1.2161	0.4294
5.17	0.58	0.3970	0.2366	0.3899	1.2161	0.4296
5.23	0.56	0.3970	0.2366	0.3900	1.2161	0.4298
5.29	0.54	0.3970	0.2366	0.3902	1.2161	0.4300
5.34	0.53	0.3970	0.2365	0.3903	1.2161	0.4302
5.40	0.51	0.3970	0.2365	0.3904	1.2161	0.4303
5.46	0.50	0.3970	0.2365	0.3905	1.2161	0.4306
5.51	0.49	0.3970	0.2364	0.3906	1.2161	0.4307
5.57	0.48	0.3970	0.2363	0.3907	1.2161	0.4308
5.63	0.46	0.3970	0.2363	0.3908	1.2161	0.4310
5.68	0.45	0.3970	0.2362	0.3909	1.2161	0.4311

Table S5. Reaction coordinate, energies relative to the separated reactants (from the Gaussian IRC), and IBO changes ($|q_i(s)$-$q_i(0)|$, from IboView) for the reaction with PhOH in DMF.

reaction coordinate	relative energy	IBO change (e^-)				
		Figure 3			Figure S4	
(bohr amu$^{1/2}$)	(kcal mol^{-1})	A	B	C	B	A
-5.76	0.16	0.0000	0.0000	0.0000	0.0000	0.0000
-5.71	0.17	0.0001	0.0001	0.0000	0.0000	0.0000
-5.65	0.18	0.0000	0.0003	0.0003	0.0001	0.0001
-5.59	0.19	0.0001	0.0004	0.0004	0.0001	0.0001
-5.53	0.20	0.0001	0.0006	0.0006	0.0001	0.0001
-5.48	0.21	0.0001	0.0007	0.0007	0.0001	0.0001
-5.42	0.23	0.0001	0.0008	0.0008	0.0002	0.0002
-5.36	0.24	0.0001	0.0010	0.0010	0.0002	0.0002
-5.30	0.25	0.0001	0.0011	0.0011	0.0003	0.0003
-5.25	0.27	0.0002	0.0012	0.0012	0.0003	0.0003
-5.19	0.28	0.0002	0.0013	0.0013	0.0004	0.0004
-5.13	0.30	0.0002	0.0015	0.0015	0.0004	0.0005
-5.07	0.32	0.0002	0.0016	0.0016	0.0006	0.0006
-5.02	0.34	0.0002	0.0017	0.0017	0.0006	0.0006
-4.96	0.36	0.0003	0.0018	0.0018	0.0008	0.0008
-4.90	0.38	0.0003	0.0019	0.0019	0.0008	0.0008
-4.84	0.40	0.0004	0.0020	0.0020	0.0010	0.0010
-4.78	0.43	0.0004	0.0022	0.0022	0.0011	0.0011
-4.73	0.45	0.0004	0.0022	0.0022	0.0012	0.0012
-4.67	0.48	0.0004	0.0024	0.0024	0.0013	0.0013
-4.61	0.50	0.0005	0.0024	0.0024	0.0015	0.0015
-4.55	0.53	0.0005	0.0026	0.0026	0.0016	0.0016
-4.50	0.56	0.0005	0.0027	0.0027	0.0017	0.0017
-4.44	0.59	0.0005	0.0028	0.0028	0.0018	0.0018
-4.38	0.63	0.0006	0.0029	0.0029	0.0020	0.0020
-4.32	0.66	0.0006	0.0030	0.0030	0.0021	0.0021
-4.27	0.69	0.0007	0.0031	0.0031	0.0023	0.0023
-4.21	0.73	0.0007	0.0032	0.0032	0.0024	0.0024
-4.15	0.77	0.0007	0.0033	0.0033	0.0026	0.0026
-4.09	0.81	0.0008	0.0034	0.0034	0.0027	0.0027
-4.04	0.85	0.0008	0.0035	0.0035	0.0029	0.0029
-3.98	0.89	0.0009	0.0036	0.0036	0.0030	0.0030
-3.92	0.93	0.0009	0.0037	0.0037	0.0032	0.0032
-3.86	0.98	0.0010	0.0038	0.0038	0.0033	0.0033
-3.81	1.02	0.0010	0.0039	0.0039	0.0034	0.0035
-3.75	1.07	0.0011	0.0041	0.0041	0.0036	0.0036
-3.69	1.12	0.0011	0.0042	0.0042	0.0037	0.0038
-3.63	1.17	0.0013	0.0043	0.0043	0.0039	0.0039
-3.57	1.23	0.0013	0.0044	0.0044	0.0040	0.0041
-3.52	1.28	0.0014	0.0045	0.0045	0.0042	0.0042
-3.46	1.33	0.0015	0.0046	0.0046	0.0043	0.0044
-3.40	1.39	0.0016	0.0048	0.0048	0.0045	0.0045
-3.34	1.45	0.0017	0.0049	0.0049	0.0047	0.0047
-3.29	1.51	0.0018	0.0050	0.0051	0.0048	0.0048
-3.23	1.57	0.0019	0.0052	0.0052	0.0049	0.0050
-3.17	1.64	0.0020	0.0053	0.0053	0.0051	0.0051
-3.11	1.70	0.0021	0.0055	0.0055	0.0053	0.0053
-3.06	1.77	0.0022	0.0056	0.0056	0.0054	0.0054

-3.00	1.84	0.0024	0.0058	0.0058	0.0056	0.0056
-2.94	1.91	0.0025	0.0060	0.0060	0.0057	0.0057
-2.88	1.98	0.0027	0.0061	0.0061	0.0059	0.0059
-2.82	2.05	0.0028	0.0063	0.0063	0.0060	0.0061
-2.77	2.13	0.0030	0.0065	0.0065	0.0062	0.0062
-2.71	2.20	0.0032	0.0067	0.0067	0.0063	0.0064
-2.65	2.28	0.0033	0.0069	0.0069	0.0065	0.0065
-2.59	2.36	0.0035	0.0072	0.0072	0.0066	0.0067
-2.54	2.45	0.0038	0.0074	0.0074	0.0068	0.0068
-2.48	2.53	0.0039	0.0076	0.0076	0.0069	0.0070
-2.42	2.62	0.0042	0.0079	0.0079	0.0071	0.0071
-2.36	2.71	0.0043	0.0082	0.0082	0.0072	0.0072
-2.31	2.80	0.0045	0.0084	0.0084	0.0074	0.0074
-2.25	2.90	0.0048	0.0088	0.0088	0.0075	0.0075
-2.19	2.99	0.0049	0.0091	0.0091	0.0076	0.0077
-2.13	3.09	0.0051	0.0094	0.0094	0.0077	0.0077
-2.07	3.20	0.0053	0.0098	0.0098	0.0078	0.0079
-2.02	3.31	0.0055	0.0102	0.0102	0.0079	0.0079
-1.96	3.42	0.0056	0.0106	0.0106	0.0080	0.0080
-1.90	3.53	0.0058	0.0110	0.0110	0.0080	0.0080
-1.84	3.65	0.0059	0.0115	0.0115	0.0080	0.0080
-1.79	3.77	0.0060	0.0120	0.0120	0.0079	0.0079
-1.73	3.90	0.0061	0.0126	0.0126	0.0079	0.0079
-1.67	4.04	0.0062	0.0132	0.0132	0.0077	0.0078
-1.61	4.18	0.0061	0.0138	0.0138	0.0076	0.0076
-1.56	4.32	0.0061	0.0145	0.0145	0.0073	0.0074
-1.50	4.48	0.0061	0.0152	0.0152	0.0071	0.0071
-1.44	4.64	0.0060	0.0159	0.0159	0.0067	0.0067
-1.38	4.81	0.0059	0.0167	0.0167	0.0063	0.0063
-1.32	5.00	0.0058	0.0176	0.0176	0.0058	0.0059
-1.27	5.19	0.0056	0.0185	0.0185	0.0053	0.0053
-1.21	5.39	0.0055	0.0194	0.0194	0.0047	0.0048
-1.15	5.61	0.0053	0.0204	0.0204	0.0041	0.0041
-1.09	5.84	0.0052	0.0215	0.0215	0.0034	0.0034
-1.04	6.09	0.0051	0.0226	0.0226	0.0027	0.0027
-0.98	6.35	0.0050	0.0237	0.0237	0.0019	0.0019
-0.92	6.62	0.0050	0.0249	0.0249	0.0011	0.0012
-0.86	6.92	0.0050	0.0262	0.0262	0.0007	0.0007
-0.81	7.23	0.0051	0.0275	0.0275	0.0011	0.0011
-0.75	7.55	0.0053	0.0292	0.0292	0.0022	0.0022
-0.69	7.89	0.0056	0.0308	0.0308	0.0032	0.0032
-0.63	8.26	0.0059	0.0316	0.0316	0.0035	0.0035
-0.58	8.65	0.0064	0.0332	0.0332	0.0045	0.0045
-0.52	9.07	0.0071	0.0350	0.0350	0.0056	0.0056
-0.46	9.52	0.0080	0.0372	0.0372	0.0070	0.0070
-0.40	10.02	0.0093	0.0404	0.0404	0.0090	0.0090
-0.35	10.59	0.0108	0.0456	0.0455	0.0124	0.0124
-0.29	11.32	0.0126	0.0532	0.0532	0.0175	0.0175
-0.23	12.26	0.0145	0.0630	0.0630	0.0239	0.0239
-0.17	13.37	0.0165	0.0746	0.0746	0.0312	0.0312
-0.12	14.62	0.0188	0.0875	0.0875	0.0392	0.0393
-0.06	15.86	0.0215	0.1017	0.1017	0.0479	0.0479
0.00	16.38	0.9659	0.1358	0.1419	0.0867	0.1714

0.06	16.01	1.1238	0.1640	0.1730	0.1059	0.2071
0.12	15.30	1.1467	0.1812	0.1901	0.1130	0.2149
0.17	14.53	1.1575	0.1966	0.2052	0.1187	0.2190
0.23	13.81	1.1630	0.2112	0.2193	0.1239	0.2222
0.29	13.21	1.1631	0.2246	0.2323	0.1286	0.2249
0.35	12.79	1.1631	0.2356	0.2430	0.1327	0.2271
0.40	12.50	1.1619	0.2423	0.2495	0.1353	0.2286
0.46	12.27	1.1627	0.2454	0.2524	0.1367	0.2295
0.52	12.06	1.1622	0.2473	0.2543	0.1378	0.2302
0.58	11.87	1.1625	0.2489	0.2559	0.1387	0.2309
0.63	11.70	1.1623	0.2504	0.2574	0.1397	0.2316
0.69	11.53	1.1620	0.2519	0.2589	0.1406	0.2322
0.75	11.38	1.1598	0.2532	0.2602	0.1415	0.2329
0.81	11.24	1.1596	0.2546	0.2615	0.1424	0.2335
0.86	11.11	1.1599	0.2558	0.2628	0.1432	0.2342
0.92	10.98	1.1599	0.2571	0.2640	0.1441	0.2348
0.98	10.86	1.1612	0.2583	0.2652	0.1448	0.2354
1.04	10.76	1.1600	0.2594	0.2664	0.1456	0.2360
1.09	10.65	1.1608	0.2605	0.2675	0.1464	0.2365
1.15	10.56	1.1619	0.2616	0.2685	0.1471	0.2370
1.21	10.47	1.1620	0.2627	0.2696	0.1478	0.2376
1.27	10.38	1.1621	0.2637	0.2706	0.1485	0.2380
1.33	10.31	1.1622	0.2647	0.2716	0.1491	0.2385
1.38	10.23	1.1622	0.2657	0.2725	0.1497	0.2389
1.44	10.16	1.1623	0.2666	0.2734	0.1503	0.2393
1.50	10.10	1.1625	0.2675	0.2743	0.1509	0.2397
1.56	10.04	1.1624	0.2683	0.2751	0.1514	0.2400
1.61	9.98	1.1625	0.2692	0.2760	0.1519	0.2403
1.67	9.93	1.1627	0.2699	0.2767	0.1523	0.2406
1.73	9.88	1.1626	0.2707	0.2774	0.1527	0.2409
1.79	9.83	1.1627	0.2714	0.2781	0.1531	0.2411
1.84	9.78	1.1626	0.2720	0.2788	0.1534	0.2413
1.90	9.74	1.1627	0.2726	0.2794	0.1538	0.2414
1.96	9.70	1.1628	0.2732	0.2799	0.1540	0.2416
2.02	9.66	1.1628	0.2737	0.2804	0.1543	0.2417
2.08	9.62	1.1628	0.2742	0.2809	0.1545	0.2418
2.13	9.58	1.1628	0.2747	0.2814	0.1547	0.2418
2.19	9.54	1.1629	0.2751	0.2818	0.1548	0.2419
2.25	9.51	1.1629	0.2754	0.2821	0.1550	0.2419
2.31	9.48	1.1629	0.2758	0.2824	0.1551	0.2419
2.36	9.44	1.1629	0.2761	0.2827	0.1552	0.2418
2.42	9.41	1.1633	0.2764	0.2830	0.1553	0.2418
2.48	9.38	1.1633	0.2766	0.2833	0.1553	0.2418
2.54	9.35	1.1633	0.2768	0.2835	0.1554	0.2417
2.59	9.33	1.1633	0.2770	0.2837	0.1554	0.2416
2.65	9.30	1.1636	0.2772	0.2839	0.1554	0.2415
2.71	9.27	1.1637	0.2774	0.2840	0.1555	0.2414
2.77	9.25	1.1628	0.2775	0.2842	0.1555	0.2413
2.83	9.22	1.1632	0.2777	0.2843	0.1555	0.2412
2.88	9.20	1.1632	0.2778	0.2844	0.1554	0.2411
2.94	9.17	1.1634	0.2779	0.2845	0.1554	0.2410
3.00	9.15	1.1635	0.2780	0.2847	0.1554	0.2409
3.06	9.13	1.1635	0.2782	0.2848	0.1554	0.2407

3.11	9.11	1.1635	0.2783	0.2848	0.1554	0.2406
3.17	9.09	1.1635	0.2783	0.2849	0.1554	0.2405
3.23	9.07	1.1635	0.2784	0.2850	0.1553	0.2404
3.29	9.05	1.1634	0.2785	0.2851	0.1553	0.2402
3.34	9.03	1.1634	0.2786	0.2852	0.1553	0.2401
3.40	9.01	1.1634	0.2787	0.2853	0.1553	0.2400
3.46	8.99	1.1635	0.2788	0.2853	0.1552	0.2399
3.52	8.97	1.1635	0.2789	0.2854	0.1552	0.2397
3.58	8.96	1.1635	0.2789	0.2855	0.1552	0.2396
3.63	8.94	1.1635	0.2790	0.2856	0.1551	0.2395
3.69	8.92	1.1636	0.2791	0.2857	0.1551	0.2394
3.75	8.91	1.1636	0.2792	0.2858	0.1551	0.2393
3.81	8.89	1.1636	0.2793	0.2858	0.1551	0.2392
3.86	8.88	1.1636	0.2794	0.2859	0.1551	0.2391
3.92	8.86	1.1636	0.2795	0.2860	0.1550	0.2390
3.98	8.85	1.1635	0.2796	0.2861	0.1550	0.2389
4.04	8.83	1.1635	0.2797	0.2862	0.1550	0.2388
4.09	8.82	1.1635	0.2798	0.2863	0.1550	0.2387
4.15	8.80	1.1636	0.2799	0.2864	0.1549	0.2387
4.21	8.79	1.1636	0.2800	0.2865	0.1549	0.2386
4.27	8.77	1.1636	0.2801	0.2866	0.1549	0.2385
4.33	8.76	1.1636	0.2802	0.2867	0.1549	0.2385
4.38	8.75	1.1636	0.2803	0.2868	0.1549	0.2384
4.44	8.73	1.1636	0.2805	0.2870	0.1549	0.2384
4.50	8.72	1.1636	0.2806	0.2871	0.1549	0.2383
4.56	8.71	1.1636	0.2807	0.2872	0.1549	0.2383
4.61	8.69	1.1633	0.2808	0.2873	0.1550	0.2382
4.67	8.68	1.1633	0.2809	0.2874	0.1550	0.2382
4.73	8.67	1.1636	0.2810	0.2875	0.1550	0.2382
4.79	8.65	1.1636	0.2811	0.2876	0.1550	0.2382
4.84	8.64	1.1636	0.2812	0.2877	0.1551	0.2382
4.90	8.63	1.1633	0.2813	0.2878	0.1551	0.2382
4.96	8.61	1.1633	0.2814	0.2878	0.1551	0.2381
5.02	8.60	1.1633	0.2815	0.2879	0.1552	0.2381
5.07	8.59	1.1633	0.2816	0.2880	0.1552	0.2381
5.13	8.58	1.1633	0.2817	0.2881	0.1553	0.2381
5.19	8.57	1.1633	0.2818	0.2882	0.1553	0.2382
5.25	8.55	1.1633	0.2818	0.2883	0.1554	0.2382
5.31	8.54	1.1633	0.2819	0.2884	0.1554	0.2382
5.36	8.53	1.1633	0.2820	0.2885	0.1555	0.2382
5.42	8.52	1.1633	0.2821	0.2886	0.1555	0.2382
5.48	8.51	1.1633	0.2822	0.2886	0.1556	0.2382
5.54	8.50	1.1633	0.2823	0.2887	0.1556	0.2382
5.59	8.49	1.1633	0.2824	0.2888	0.1557	0.2382
5.65	8.48	1.1634	0.2824	0.2889	0.1557	0.2383
5.71	8.47	1.1634	0.2825	0.2890	0.1558	0.2383
5.77	8.46	1.1634	0.2826	0.2890	0.1558	0.2383

8. Dipole Projection

Table S6: Reaction coordinate dipole moments components along the x, y, and z axes and projected dipole moments (from ORCA) for the reaction with CHD in DMF.

reaction coordinate (bohr amu$^{1/2}$)	Dipole moment (D)			projected dipole moment (D)
	x	y	z	
-5.68	-5.1813	3.2024	-0.2462	14.77
-5.63	-5.1833	3.2020	-0.2471	14.77
-5.57	-5.1859	3.2009	-0.2477	14.78
-5.51	-5.1889	3.2000	-0.2485	14.79
-5.46	-5.1921	3.1981	-0.2492	14.79
-5.40	-5.1962	3.1965	-0.2501	14.80
-5.34	-5.2002	3.1941	-0.2508	14.81
-5.29	-5.2051	3.1920	-0.2519	14.82
-5.23	-5.2096	3.1895	-0.2524	14.83
-5.17	-5.2144	3.1869	-0.2535	14.84
-5.12	-5.2192	3.1844	-0.2539	14.85
-5.06	-5.2241	3.1814	-0.2549	14.86
-5.00	-5.2292	3.1788	-0.2553	14.87
-4.95	-5.2342	3.1755	-0.2562	14.88
-4.89	-5.2395	3.1729	-0.2565	14.89
-4.83	-5.2442	3.1698	-0.2574	14.90
-4.78	-5.2493	3.1671	-0.2577	14.91
-4.72	-5.2540	3.1640	-0.2583	14.91
-4.66	-5.2589	3.1613	-0.2587	14.92
-4.61	-5.2634	3.1583	-0.2592	14.93
-4.55	-5.2683	3.1556	-0.2596	14.94
-4.49	-5.2728	3.1526	-0.2601	14.95
-4.43	-5.2775	3.1499	-0.2604	14.96
-4.38	-5.2819	3.1468	-0.2609	14.97
-4.32	-5.2866	3.1442	-0.2612	14.98
-4.26	-5.2909	3.1412	-0.2617	14.99
-4.21	-5.2955	3.1386	-0.2620	14.99
-4.15	-5.2994	3.1355	-0.2626	15.00
-4.09	-5.3038	3.1328	-0.2629	15.01
-4.04	-5.3077	3.1298	-0.2634	15.02
-3.98	-5.3119	3.1270	-0.2638	15.03
-3.92	-5.3157	3.1241	-0.2642	15.03
-3.87	-5.3197	3.1210	-0.2646	15.04
-3.81	-5.3234	3.1182	-0.2651	15.05
-3.75	-5.3273	3.1147	-0.2656	15.05
-3.70	-5.3309	3.1120	-0.2661	15.06
-3.64	-5.3349	3.1081	-0.2668	15.07
-3.58	-5.3384	3.1053	-0.2671	15.07
-3.53	-5.3420	3.1013	-0.2679	15.08
-3.47	-5.3454	3.0981	-0.2682	15.09
-3.41	-5.3487	3.0942	-0.2690	15.09
-3.35	-5.3519	3.0905	-0.2695	15.10
-3.30	-5.3549	3.0865	-0.2702	15.10
-3.24	-5.3578	3.0825	-0.2708	15.11
-3.18	-5.3605	3.0783	-0.2714	15.11
-3.13	-5.3633	3.0740	-0.2721	15.11

-3.07	-5.3657	3.0695	-0.2728	15.12
-3.01	-5.3680	3.0649	-0.2736	15.12
-2.96	-5.3701	3.0602	-0.2743	15.12
-2.90	-5.3721	3.0554	-0.2751	15.12
-2.84	-5.3738	3.0503	-0.2759	15.12
-2.79	-5.3754	3.0452	-0.2767	15.13
-2.73	-5.3768	3.0401	-0.2775	15.13
-2.67	-5.3778	3.0348	-0.2784	15.12
-2.61	-5.3786	3.0292	-0.2793	15.12
-2.56	-5.3792	3.0237	-0.2802	15.12
-2.50	-5.3794	3.0181	-0.2812	15.12
-2.44	-5.3794	3.0124	-0.2821	15.11
-2.39	-5.3790	3.0065	-0.2831	15.11
-2.33	-5.3784	3.0006	-0.2841	15.11
-2.27	-5.3774	2.9947	-0.2851	15.10
-2.22	-5.3760	2.9886	-0.2861	15.09
-2.16	-5.3742	2.9825	-0.2871	15.08
-2.10	-5.3720	2.9762	-0.2881	15.07
-2.05	-5.3695	2.9700	-0.2890	15.06
-1.99	-5.3664	2.9637	-0.2900	15.05
-1.93	-5.3630	2.9574	-0.2909	15.04
-1.88	-5.3588	2.9508	-0.2918	15.03
-1.82	-5.3545	2.9445	-0.2926	15.01
-1.76	-5.3488	2.9376	-0.2934	14.99
-1.71	-5.3436	2.9312	-0.2941	14.97
-1.65	-5.3366	2.9241	-0.2947	14.95
-1.59	-5.3289	2.9169	-0.2952	14.93
-1.54	-5.3205	2.9096	-0.2955	14.90
-1.48	-5.3105	2.9018	-0.2958	14.87
-1.42	-5.2995	2.8939	-0.2958	14.84
-1.36	-5.2872	2.8856	-0.2956	14.80
-1.31	-5.2733	2.8768	-0.2953	14.76
-1.25	-5.2578	2.8674	-0.2947	14.71
-1.19	-5.2407	2.8576	-0.2939	14.66
-1.14	-5.2216	2.8472	-0.2929	14.60
-1.08	-5.2004	2.8359	-0.2916	14.54
-1.02	-5.1771	2.8240	-0.2901	14.48
-0.97	-5.1513	2.8112	-0.2883	14.40
-0.91	-5.1231	2.7975	-0.2862	14.32
-0.85	-5.0921	2.7828	-0.2839	14.23
-0.80	-5.0582	2.7670	-0.2812	14.13
-0.74	-5.0211	2.7499	-0.2781	14.03
-0.68	-4.9805	2.7315	-0.2748	13.91
-0.63	-4.9362	2.7116	-0.2710	13.79
-0.57	-4.8876	2.6900	-0.2668	13.65
-0.51	-4.8342	2.6665	-0.2622	13.50
-0.45	-4.7750	2.6405	-0.2571	13.34
-0.40	-4.7078	2.6114	-0.2512	13.15
-0.34	-4.6285	2.5770	-0.2445	12.93
-0.28	-4.5288	2.5340	-0.2362	12.66
-0.23	-4.3993	2.4778	-0.2262	12.30
-0.17	-4.1849	2.3898	-0.2063	11.72
-0.11	-3.5192	2.1500	-0.1185	9.93

-0.06	-2.8947	1.9224	-0.0383	8.24
0.00	-2.3457	1.7181	0.0287	6.77
0.06	-1.8941	1.5443	0.0789	5.55
0.11	-1.5465	1.4032	0.1111	4.61
0.17	-1.2977	1.2931	0.1262	3.94
0.23	-1.1349	1.2100	0.1263	3.49
0.28	-1.0420	1.1490	0.1141	3.24
0.34	-1.0036	1.1052	0.0927	3.13
0.40	-1.0060	1.0748	0.0645	3.13
0.46	-1.0376	1.0544	0.0319	3.21
0.51	-1.0887	1.0413	-0.0027	3.34
0.57	-1.1506	1.0334	-0.0373	3.50
0.63	-1.2135	1.0288	-0.0690	3.66
0.68	-1.2664	1.0254	-0.0941	3.80
0.74	-1.3017	1.0220	-0.1103	3.88
0.80	-1.3233	1.0187	-0.1202	3.94
0.85	-1.3388	1.0158	-0.1270	3.97
0.91	-1.3521	1.0139	-0.1327	4.00
0.97	-1.3653	1.0129	-0.1379	4.04
1.02	-1.3785	1.0131	-0.1428	4.07
1.08	-1.3919	1.0144	-0.1476	4.10
1.14	-1.4054	1.0169	-0.1521	4.14
1.19	-1.4189	1.0206	-0.1564	4.17
1.25	-1.4323	1.0253	-0.1606	4.21
1.31	-1.4455	1.0312	-0.1646	4.24
1.36	-1.4582	1.0381	-0.1684	4.28
1.42	-1.4704	1.0459	-0.1721	4.31
1.48	-1.4821	1.0545	-0.1758	4.35
1.54	-1.4926	1.0638	-0.1792	4.38
1.59	-1.5034	1.0737	-0.1831	4.41
1.65	-1.5110	1.0841	-0.1859	4.44
1.71	-1.5216	1.0952	-0.1906	4.47
1.76	-1.5275	1.1069	-0.1935	4.49
1.82	-1.5339	1.1186	-0.1971	4.52
1.88	-1.5402	1.1302	-0.2010	4.54
1.93	-1.5456	1.1419	-0.2047	4.56
1.99	-1.5507	1.1535	-0.2086	4.58
2.05	-1.5552	1.1656	-0.2124	4.60
2.10	-1.5593	1.1775	-0.2163	4.62
2.16	-1.5630	1.1900	-0.2199	4.64
2.22	-1.5666	1.2019	-0.2237	4.65
2.27	-1.5699	1.2145	-0.2273	4.67
2.33	-1.5730	1.2266	-0.2309	4.69
2.39	-1.5761	1.2390	-0.2343	4.70
2.44	-1.5791	1.2510	-0.2376	4.72
2.50	-1.5820	1.2633	-0.2407	4.73
2.56	-1.5849	1.2750	-0.2436	4.75
2.61	-1.5879	1.2868	-0.2463	4.76
2.67	-1.5909	1.2980	-0.2490	4.77
2.73	-1.5939	1.3092	-0.2513	4.79
2.79	-1.5970	1.3198	-0.2536	4.80
2.84	-1.6001	1.3304	-0.2555	4.81
2.90	-1.6032	1.3402	-0.2575	4.83

2.96	-1.6064	1.3502	-0.2590	4.84
3.01	-1.6096	1.3593	-0.2607	4.85
3.07	-1.6127	1.3687	-0.2619	4.86
3.13	-1.6160	1.3770	-0.2633	4.87
3.18	-1.6192	1.3857	-0.2642	4.88
3.24	-1.6227	1.3934	-0.2654	4.89
3.30	-1.6262	1.4013	-0.2662	4.90
3.35	-1.6298	1.4084	-0.2672	4.91
3.41	-1.6335	1.4155	-0.2679	4.92
3.47	-1.6373	1.4220	-0.2688	4.93
3.52	-1.6412	1.4283	-0.2696	4.94
3.58	-1.6451	1.4339	-0.2705	4.95
3.64	-1.6494	1.4395	-0.2713	4.96
3.70	-1.6533	1.4443	-0.2723	4.97
3.75	-1.6580	1.4492	-0.2733	4.99
3.81	-1.6619	1.4533	-0.2744	4.99
3.87	-1.6670	1.4575	-0.2757	5.01
3.92	-1.6710	1.4609	-0.2770	5.02
3.98	-1.6764	1.4645	-0.2786	5.03
4.04	-1.6805	1.4674	-0.2799	5.04
4.09	-1.6860	1.4704	-0.2820	5.05
4.15	-1.6904	1.4728	-0.2834	5.06
4.21	-1.6957	1.4751	-0.2858	5.07
4.26	-1.7005	1.4772	-0.2874	5.08
4.32	-1.7057	1.4790	-0.2898	5.09
4.38	-1.7106	1.4808	-0.2916	5.11
4.43	-1.7156	1.4822	-0.2941	5.12
4.49	-1.7205	1.4838	-0.2960	5.13
4.55	-1.7256	1.4849	-0.2985	5.14
4.60	-1.7303	1.4864	-0.3004	5.15
4.66	-1.7353	1.4875	-0.3029	5.16
4.72	-1.7399	1.4888	-0.3048	5.17
4.78	-1.7448	1.4899	-0.3073	5.18
4.83	-1.7491	1.4912	-0.3092	5.19
4.89	-1.7539	1.4923	-0.3116	5.20
4.95	-1.7579	1.4936	-0.3135	5.21
5.00	-1.7625	1.4946	-0.3158	5.22
5.06	-1.7663	1.4957	-0.3176	5.23
5.12	-1.7706	1.4966	-0.3198	5.24
5.17	-1.7740	1.4974	-0.3216	5.25
5.23	-1.7782	1.4980	-0.3237	5.25
5.29	-1.7811	1.4986	-0.3254	5.26
5.34	-1.7853	1.4989	-0.3275	5.27
5.40	-1.7874	1.4991	-0.3290	5.27
5.46	-1.7919	1.4990	-0.3309	5.28
5.51	-1.7936	1.4989	-0.3325	5.29
5.57	-1.7972	1.4984	-0.3341	5.30
5.63	-1.7991	1.4981	-0.3357	5.30
5.68	-1.8014	1.4974	-0.3369	5.30

**Table S7:** Reaction coordinate, dipole moments components along the x, y, and z axes and projected dipole moments (from ORCA) for the reaction with PhOH in DMF.				
**reaction coordinate (bohr amu$^{1/2}$)**	**Dipole moment (D)**			**project dipole moment (D)**
	**x**	**y**	**z**	
-5.76	-8.2030	1.4712	-0.9124	21.31
-5.71	-8.2019	1.4710	-0.9115	21.31
-5.65	-8.2005	1.4707	-0.9107	21.30
-5.59	-8.1998	1.4707	-0.9098	21.30
-5.53	-8.1985	1.4706	-0.9089	21.30
-5.48	-8.1981	1.4706	-0.9079	21.30
-5.42	-8.1970	1.4707	-0.9069	21.29
-5.36	-8.1968	1.4710	-0.9060	21.29
-5.30	-8.1957	1.4711	-0.9049	21.29
-5.25	-8.1958	1.4715	-0.9040	21.29
-5.19	-8.1951	1.4718	-0.9029	21.29
-5.13	-8.1955	1.4722	-0.9020	21.29
-5.07	-8.1949	1.4726	-0.9010	21.29
-5.02	-8.1957	1.4730	-0.9001	21.29
-4.96	-8.1955	1.4735	-0.8991	21.29
-4.90	-8.1963	1.4739	-0.8983	21.29
-4.84	-8.1965	1.4744	-0.8973	21.29
-4.78	-8.1976	1.4748	-0.8965	21.29
-4.73	-8.1980	1.4754	-0.8956	21.29
-4.67	-8.1993	1.4757	-0.8948	21.30
-4.61	-8.1999	1.4763	-0.8939	21.30
-4.55	-8.2016	1.4766	-0.8933	21.30
-4.50	-8.2024	1.4772	-0.8923	21.31
-4.44	-8.2045	1.4773	-0.8919	21.31
-4.38	-8.2054	1.4778	-0.8910	21.31
-4.32	-8.2081	1.4778	-0.8907	21.32
-4.27	-8.2090	1.4783	-0.8898	21.32
-4.21	-8.2119	1.4784	-0.8895	21.33
-4.15	-8.2132	1.4787	-0.8888	21.33
-4.09	-8.2160	1.4789	-0.8884	21.34
-4.04	-8.2177	1.4791	-0.8878	21.34
-3.98	-8.2205	1.4793	-0.8874	21.35
-3.92	-8.2224	1.4796	-0.8868	21.35
-3.86	-8.2252	1.4798	-0.8864	21.36
-3.81	-8.2274	1.4801	-0.8859	21.37
-3.75	-8.2302	1.4804	-0.8855	21.37
-3.69	-8.2326	1.4807	-0.8851	21.38
-3.63	-8.2355	1.4811	-0.8847	21.39
-3.57	-8.2381	1.4815	-0.8843	21.39
-3.52	-8.2410	1.4819	-0.8839	21.40
-3.46	-8.2437	1.4823	-0.8835	21.41
-3.40	-8.2469	1.4827	-0.8832	21.42
-3.34	-8.2497	1.4831	-0.8829	21.42
-3.29	-8.2531	1.4837	-0.8826	21.43
-3.23	-8.2561	1.4841	-0.8823	21.44
-3.17	-8.2595	1.4846	-0.8820	21.45
-3.11	-8.2628	1.4851	-0.8817	21.46
-3.06	-8.2663	1.4856	-0.8815	21.47

-3.00	-8.2697	1.4861	-0.8813	21.47
-2.94	-8.2734	1.4866	-0.8811	21.48
-2.88	-8.2770	1.4871	-0.8810	21.49
-2.82	-8.2809	1.4876	-0.8809	21.50
-2.77	-8.2848	1.4882	-0.8807	21.51
-2.71	-8.2890	1.4886	-0.8807	21.52
-2.65	-8.2932	1.4891	-0.8806	21.53
-2.59	-8.2977	1.4896	-0.8807	21.54
-2.54	-8.3021	1.4900	-0.8807	21.56
-2.48	-8.3069	1.4905	-0.8808	21.57
-2.42	-8.3117	1.4909	-0.8809	21.58
-2.36	-8.3170	1.4912	-0.8811	21.59
-2.31	-8.3223	1.4917	-0.8812	21.61
-2.25	-8.3281	1.4920	-0.8816	21.62
-2.19	-8.3340	1.4923	-0.8818	21.64
-2.13	-8.3403	1.4926	-0.8823	21.65
-2.07	-8.3471	1.4931	-0.8825	21.67
-2.02	-8.3543	1.4933	-0.8832	21.69
-1.96	-8.3623	1.4939	-0.8834	21.71
-1.90	-8.3706	1.4942	-0.8842	21.73
-1.84	-8.3796	1.4949	-0.8846	21.75
-1.79	-8.3895	1.4955	-0.8853	21.78
-1.73	-8.4001	1.4964	-0.8859	21.80
-1.67	-8.4115	1.4974	-0.8867	21.83
-1.61	-8.4239	1.4987	-0.8874	21.86
-1.56	-8.4373	1.5001	-0.8882	21.90
-1.50	-8.4515	1.5017	-0.8890	21.94
-1.44	-8.4666	1.5035	-0.8899	21.97
-1.38	-8.4828	1.5055	-0.8907	22.02
-1.32	-8.4996	1.5077	-0.8917	22.06
-1.27	-8.5173	1.5101	-0.8926	22.10
-1.21	-8.5356	1.5126	-0.8937	22.15
-1.15	-8.5545	1.5153	-0.8948	22.20
-1.09	-8.5739	1.5180	-0.8961	22.25
-1.04	-8.5938	1.5209	-0.8974	22.30
-0.98	-8.6138	1.5237	-0.8989	22.35
-0.92	-8.6341	1.5268	-0.9004	22.40
-0.86	-8.6546	1.5297	-0.9022	22.46
-0.81	-8.6759	1.5329	-0.9041	22.51
-0.75	-8.7021	1.5353	-0.9072	22.58
-0.69	-8.7284	1.5383	-0.9095	22.65
-0.63	-8.7438	1.5438	-0.9088	22.69
-0.58	-8.7669	1.5480	-0.9103	22.75
-0.52	-8.7938	1.5524	-0.9120	22.82
-0.46	-8.8265	1.5567	-0.9143	22.90
-0.40	-8.8721	1.5596	-0.9185	23.02
-0.35	-8.9389	1.5590	-0.9268	23.18
-0.29	-9.0248	1.5526	-0.9409	23.40
-0.23	-9.1128	1.5405	-0.9588	23.62
-0.17	-9.1836	1.5235	-0.9785	23.79
-0.12	-9.2200	1.5012	-0.9979	23.88
-0.06	-9.2074	1.4734	-1.0159	23.84
0.00	-3.9402	0.9434	-0.8048	10.50

0.06	-0.2960	0.5426	-0.6646	2.31
0.12	0.1555	0.4568	-0.6670	2.09
0.17	0.1796	0.4156	-0.6886	2.09
0.23	0.0946	0.3874	-0.7144	2.08
0.29	-0.0231	0.3655	-0.7401	2.10
0.35	-0.1351	0.3500	-0.7613	2.16
0.40	-0.2092	0.3432	-0.7731	2.21
0.46	-0.2447	0.3427	-0.7771	2.25
0.52	-0.2679	0.3443	-0.7790	2.27
0.58	-0.2881	0.3465	-0.7807	2.29
0.63	-0.3076	0.3494	-0.7824	2.31
0.69	-0.3269	0.3527	-0.7843	2.34
0.75	-0.3461	0.3564	-0.7862	2.36
0.81	-0.3653	0.3604	-0.7882	2.39
0.86	-0.3842	0.3647	-0.7901	2.42
0.92	-0.4027	0.3690	-0.7920	2.45
0.98	-0.4209	0.3734	-0.7938	2.47
1.04	-0.4387	0.3779	-0.7956	2.50
1.09	-0.4560	0.3823	-0.7972	2.53
1.15	-0.4730	0.3866	-0.7989	2.56
1.21	-0.4894	0.3907	-0.8005	2.58
1.27	-0.5054	0.3947	-0.8020	2.61
1.33	-0.5208	0.3984	-0.8035	2.64
1.38	-0.5358	0.4019	-0.8049	2.66
1.44	-0.5502	0.4051	-0.8063	2.69
1.50	-0.5641	0.4082	-0.8076	2.71
1.56	-0.5774	0.4108	-0.8089	2.73
1.61	-0.5902	0.4133	-0.8101	2.76
1.67	-0.6024	0.4155	-0.8113	2.78
1.73	-0.6140	0.4175	-0.8124	2.80
1.79	-0.6249	0.4193	-0.8134	2.82
1.84	-0.6353	0.4209	-0.8144	2.84
1.90	-0.6452	0.4224	-0.8153	2.85
1.96	-0.6544	0.4236	-0.8161	2.87
2.02	-0.6630	0.4247	-0.8168	2.88
2.08	-0.6710	0.4257	-0.8174	2.90
2.13	-0.6785	0.4266	-0.8180	2.91
2.19	-0.6855	0.4274	-0.8184	2.92
2.25	-0.6919	0.4281	-0.8188	2.93
2.31	-0.6979	0.4287	-0.8191	2.94
2.36	-0.7034	0.4293	-0.8193	2.95
2.42	-0.7086	0.4297	-0.8194	2.96
2.48	-0.7133	0.4302	-0.8195	2.97
2.54	-0.7177	0.4305	-0.8194	2.98
2.59	-0.7218	0.4309	-0.8194	2.98
2.65	-0.7256	0.4312	-0.8192	2.99
2.71	-0.7292	0.4315	-0.8190	3.00
2.77	-0.7325	0.4316	-0.8188	3.00
2.83	-0.7356	0.4319	-0.8185	3.00
2.88	-0.7385	0.4320	-0.8181	3.01
2.94	-0.7414	0.4321	-0.8178	3.01
3.00	-0.7440	0.4322	-0.8173	3.02
3.06	-0.7465	0.4323	-0.8169	3.02

3.11	-0.7490	0.4323	-0.8163	3.02
3.17	-0.7513	0.4323	-0.8158	3.03
3.23	-0.7536	0.4324	-0.8152	3.03
3.29	-0.7558	0.4323	-0.8146	3.03
3.34	-0.7579	0.4323	-0.8139	3.03
3.40	-0.7600	0.4322	-0.8133	3.04
3.46	-0.7621	0.4321	-0.8126	3.04
3.52	-0.7641	0.4319	-0.8119	3.04
3.58	-0.7662	0.4318	-0.8111	3.04
3.63	-0.7681	0.4316	-0.8104	3.04
3.69	-0.7701	0.4313	-0.8097	3.04
3.75	-0.7722	0.4310	-0.8089	3.05
3.81	-0.7742	0.4306	-0.8082	3.05
3.86	-0.7762	0.4302	-0.8075	3.05
3.92	-0.7781	0.4297	-0.8067	3.05
3.98	-0.7802	0.4292	-0.8060	3.05
4.04	-0.7822	0.4286	-0.8053	3.05
4.09	-0.7842	0.4281	-0.8046	3.06
4.15	-0.7862	0.4274	-0.8039	3.06
4.21	-0.7881	0.4267	-0.8031	3.06
4.27	-0.7901	0.4260	-0.8023	3.06
4.33	-0.7919	0.4253	-0.8015	3.06
4.38	-0.7938	0.4247	-0.8006	3.06
4.44	-0.7955	0.4240	-0.7996	3.06
4.50	-0.7973	0.4233	-0.7986	3.06
4.56	-0.7989	0.4226	-0.7975	3.06
4.61	-0.8006	0.4219	-0.7963	3.06
4.67	-0.8022	0.4213	-0.7951	3.06
4.73	-0.8037	0.4206	-0.7938	3.06
4.79	-0.8053	0.4200	-0.7925	3.06
4.84	-0.8067	0.4193	-0.7911	3.06
4.90	-0.8081	0.4186	-0.7896	3.06
4.96	-0.8096	0.4179	-0.7882	3.06
5.02	-0.8110	0.4172	-0.7867	3.06
5.07	-0.8123	0.4165	-0.7851	3.06
5.13	-0.8137	0.4158	-0.7835	3.06
5.19	-0.8150	0.4150	-0.7819	3.06
5.25	-0.8163	0.4143	-0.7803	3.06
5.31	-0.8176	0.4134	-0.7786	3.06
5.36	-0.8188	0.4125	-0.7769	3.05
5.42	-0.8201	0.4117	-0.7751	3.05
5.48	-0.8214	0.4108	-0.7734	3.05
5.54	-0.8227	0.4099	-0.7716	3.05
5.59	-0.8239	0.4090	-0.7697	3.05
5.65	-0.8251	0.4080	-0.7679	3.05
5.71	-0.8264	0.4070	-0.7660	3.05
5.77	-0.8277	0.4060	-0.7642	3.04

9. References

[1] Gaussian 16, Revision C.01, M. J. Frisch, G. W. Trucks, H. B. Schlegel, G. E. Scuseria, M. A. Robb, J. R. Cheeseman, G. Scalmani, V. Barone, G. A. Petersson, H. Nakatsuji, X. Li, M. Caricato, A. V. Marenich, J. Bloino, B. G. Janesko, R. Gomperts, B. Mennucci, H. P. Hratchian, J. V. Ortiz, A. F. Izmaylov, J. L. Sonnenberg, D. Williams-Young, F. Ding, F. Lipparini, F. Egidi, J. Goings, B. Peng, A. Petrone, T. Henderson, D. Ranasinghe, V. G. Zakrzewski, J. Gao, N. Rega, G. Zheng, W. Liang, M. Hada, M. Ehara, K. Toyota, R. Fukuda, J. Hasegawa, M. Ishida, T. Nakajima, Y. Honda, O. Kitao, H. Nakai, T. Vreven, K. Throssell, J. A. Montgomery, Jr., J. E. Peralta, F. Ogliaro, M. J. Bearpark, J. J. Heyd, E. N. Brothers, K. N. Kudin, V. N. Staroverov, T. A. Keith, R. Kobayashi, J. Normand, K. Raghavachari, A. P. Rendell, J. C. Burant, S. S. Iyengar, J. Tomasi, M. Cossi, J. M. Millam, M. Klene, C. Adamo, R. Cammi, J. W. Ochterski, R. L. Martin, K. Morokuma, O. Farkas, J. B. Foresman, and D. J. Fox, Gaussian, Inc., Wallingford CT, 2016.

[2] C. Adamo, M. Cossi, V. Barone, *J. Mol. Struct.: THEOCHEM* **1999**, *493*, 145-157.

[3] F. Weigend, R. Ahlrichs, *Phys. Chem. Chem. Phys.* **2005**, *7*, 3297-3305.

[4] B. P. Pritchard, D. Altarawy, B. Didier, T. D. Gibson, T. L. Windus, *J. Chem. Inf. Model.* **2019**, *59*, 4814-4820.

[5] D. Andrae, U. Häußermann, M. Dolg, H. Stoll, H. Preuß, *Theor. Chim. Acta* **1990**, *77*, 123-141.

[6] S. Grimme, J. Antony, S. Ehrlich, H. Krieg, *J. Chem. Phys.* **2010**, *132*, 154104.

[7] S. Grimme, S. Ehrlich, L. Goerigk, *J. Comput. Chem.* **2011**, *32*, 1456-1465.

[8] a) S. Miertuš, E. Scrocco, J. Tomasi, *Chem. Phys.* **1981**, *55*, 117-129; b) S. Miertuš, J. Tomasi, *Chem. Phys.* **1982**, *65*, 239-245; c) J. L. Pascual-ahuir, E. Silla, I. Tuñon, *J. Comput. Chem.* **1994**, *15*, 1127-1138.

[9] V. S. Bryantsev, M. S. Diallo, W. A. G. III, *J. Phys. Chem. B* **2008**, *112*, 9709-9719.

[10] a) T. Ziegler, A. Rauk, E. J. Baerends, *Theor. Chim. Acta* **1977**, *43*, 261-271; b) L. Noodleman, J. G. Norman, *J. Chem. Phys.* **1979**, *1979*, 4903-4906; c) K. Yamaguchi, F. Jensen, A. Dorigo, K. N. Houk, *Chem. Phys. Lett.* **1988**, *149*, 537-542; d) L. Noodleman and D. A. Case, in Advances in Inorganic Chemistry, ed. C. Richard, Academic Press, 1992, vol. Volume 1938, pp. 1423-1470; e) M. H. Lim, S. E. Worthington, F. J. Dulles and C. J. Cramer, in Chemical Applications of Density Functional Theory, American Chemical Society, 1996, vol. 1629, ch. 1927, pp. 1402-1422; f) H. Isobe, Y. Takano, Y. Kitagawa, T. Kawakami, S. Yamanaka, K. Yamaguchi, K. N. Houk, *Molecular Physics* **2002**, *100*, 717-727; g) S. M. Huber, M. Z. Ertem, F. Aquilante, L. Gagliardi, W. B. Tolman, C. J. Cramer, *Chem. Eur. J.* **2009**, *15*, 4886-4895; h) J. E. M. N. Klein, B. Dereli, L. Que, C. J. Cramer, *Chem. Comm.* **2016**, *52*, 10509-10512.

[11] G. Knizia, J. E. M. N. Klein, *Angew. Chem. Int. Ed.* **2015**, *54*, 5518-5522.

[12] a) F. Neese, *WIREs: Comput. Mol. Sci.* **2012**, *2*, 73-78; b) F. Neese, *WIREs: Comput. Mol. Sci.* **2018**, *8*, 1327.

[13] V. Barone, M. Cossi, *J. Phys. Chem. A* **1998**, *102*, 1995-2001.

[14] F. Weigend, *Phys. Chem. Chem. Phys.* **2006**, *8*, 1057-1065.

[15] G. Knizia, *J. Chem. Theory Comput.* **2013**, *9*, 4834-4843.

[16] M. Mandal, C. E. Elwell, C. J. Bouchey, T. J. Zerk, W. B. Tolman, C. J. Cramer, *J. Am. Chem. Soc.* **2019**, *141*, 17236-17244.

[17] a) GAMESS-UK is a package of ab initio programs. See: "http://www.computingforscience.com/gamess-uk/index.shtml", M.F. Guest, I. J. Bush, H.J.J. van Dam, P. Sherwood, J.M.H. Thomas, J.H. van Lenthe, R.W.A Havenith, J. Kendrick, "The GAMESS-UK electronic structure package: algorithms, developments and applications", Molecular Physics, Vol. 103, No. 106-108, 120 March-120 April 2005, 2719-2747; b) P. J. Knowles, H.-J. Werner, *Chem. Phys. Lett.* **1985**, *115*, 259-267; c) P. J. Knowles, G. J. Sexton, N. C. Handy, *Chem. Phys.* **1982**, *72*, 337-347.

[18] J. M. Bofill, P. Pulay, *J. Chem. Phys.* **1989**, *90*, 3637-3646.
[19] a) E. Ramos-Cordoba, E. Matito, I. Mayer, P. Salvador, *J. Chem. Theory Comput.* **2012**, *8*, 1270-1279; b) V. N. Staroverov, E. R. Davidson, *Int. J. Quantum Chem.* **2000**, *77*, 316-323; c) V. N. Staroverov, E. R. Davidson, *J. Am. Chem. Soc.* **2000**, *122*, 186-187.
[20] M. Lovisari, A. R. McDonald, *Inorg. Chem.* **2020**, *59*, 3659-3665.
[21] D. Dhar, G. M. Yee, T. F. Markle, J. M. Mayer, W. B. Tolman, *Chem. Sci.* **2017**, *8*, 1075-1085.
